# Risk factors for preterm birth: an umbrella review of meta-analyses of observational studies

**DOI:** 10.1186/s12916-023-03171-4

**Published:** 2023-12-13

**Authors:** Ioannis Mitrogiannis, Evangelos Evangelou, Athina Efthymiou, Theofilos Kanavos, Effrosyni Birbas, George Makrydimas, Stefania Papatheodorou

**Affiliations:** 1 Department of Obstetrics & Gynecology, General Hospital of Arta, 47100 Arta, Greece; 2 Department of Epidemiology and Biostatistics, Imperial College London, London, SW7 2AZ UK; 3 Department of Hygiene and Epidemiology, University of Ioannina Medical School, 45110 Ioannina, Greece; 4 Harris Birthright Research Centre for Fetal Medicine, King’s College London, London, SE5 8BB UK; 5 Department of Women and Children Health, NHS Foundation Trust, Guy’s and St Thomas, London, SE1 7EH UK; 6 University of Ioannina Medical School, 45110 Ioannina, Greece; 7 Department of Obstetrics & Gynecology, University Hospital of Ioannina, 45110 Ioannina, Greece; 8 Department of Epidemiology, Harvard T.H. Chan School of Public Health, Boston, MA 02115 USA

**Keywords:** Pregnancy complications, Obstetrics, Umbrella review, Risk factors, Preterm birth, Preventive medicine

## Abstract

**Background:**

Preterm birth defined as delivery before 37 gestational weeks is a leading cause of neonatal and infant morbidity and mortality. The aim of this study is to summarize the evidence from meta-analyses of observational studies on risk factors associated with PTB, evaluate whether there are indications of biases in this literature, and identify which of the previously reported associations are supported by robust evidence.

**Methods:**

We searched PubMed and Scopus until February 2021, in order to identify meta-analyses examining associations between risk factors and PTB. For each meta-analysis, we estimated the summary effect size, the 95% confidence interval, the 95% prediction interval, the between-study heterogeneity, evidence of small-study effects, and evidence of excess-significance bias. Evidence was graded as robust, highly suggestive, suggestive, and weak.

**Results:**

Eighty-five eligible meta-analyses were identified, which included 1480 primary studies providing data on 166 associations, covering a wide range of comorbid diseases, obstetric and medical history, drugs, exposure to environmental agents, infections, and vaccines. Ninety-nine (59.3%) associations were significant at *P* < 0.05, while 41 (24.7%) were significant at *P* < 10^−6^. Ninety-one (54.8%) associations had large or very large heterogeneity. Evidence for small-study effects and excess significance bias was found in 37 (22.3%) and 12 (7.2%) associations, respectively. We evaluated all associations according to prespecified criteria. Seven risk factors provided robust evidence: amphetamine exposure, isolated single umbilical artery, maternal personality disorder, sleep-disordered breathing (SDB), prior induced termination of pregnancy with vacuum aspiration (I-TOP with VA), low gestational weight gain (GWG), and interpregnancy interval (IPI) following miscarriage < 6 months.

**Conclusions:**

The results from the synthesis of observational studies suggest that seven risk factors for PTB are supported by robust evidence. Routine screening for sleep quality and mental health is currently lacking from prenatal visits and should be introduced. This assessment can promote the development and training of prediction models using robust risk factors that could improve risk stratification and guide cost-effective preventive strategies.

**Trial registration:**

PROSPERO 2021 CRD42021227296.

**Supplementary Information:**

The online version contains supplementary material available at 10.1186/s12916-023-03171-4.

## Background

Preterm birth (PTB) is defined as delivery before 37 gestational weeks and is a leading cause of infant morbidity and mortality [1–4]. It is estimated that 15 million babies are born preterm annually and the PTB rate ranges between 5 and 18% worldwide [3]. Specifically, the prevalence of PTB varies by geographic location ranging from 12 to 13% in the USA [1, 2] and from 5 to 9% in Europe [2]. Advances in neonatology and the administration of corticosteroids before birth have significantly improved the prognosis of babies born preterm [5]. Even though vigorous research was carried out over the last 40 years, which costed millions of dollars and focused on the prediction and prevention of preterm birth its incidence remains relatively unchanged [5]. The most probable explanation is that preterm birth is a syndrome, and many different causes may act synergistically to its manifestation [5].

Numerous systematic reviews and meta-analyses have assessed various, non-genetic risk factors of preterm labor. Several environmental and clinical parameters such as present pregnancy characteristics, previous pregnancy history [4], infections [6, 7], environmental exposures, pharmaceutical factors [8], and surgical interventions have been proposed as plausible factors related to PTB [9]. To date, there is no assessment of the epidemiological quality of this literature. Identifying robust risk factors for PTB should help us define a study population for specific interventions, allocate available resources effectively, allow for risk-specific treatment, and understand the mechanisms leading to PTB [1].

To our knowledge, there is no previous effort to summarize existing evidence of meta-analyses of non-genetic risk factors for PTB. We conducted an umbrella review across published meta-analyses of observational studies, including topics related to a wide range of risk factors including obstetric history, medical history, drugs, socioeconomic status indicators, and environmental and dietary risk factors, with the goal of mapping the existing evidence and critically evaluating the reported associations. We applied stringent criteria to assess potential systematic biases.

## Methods

We conducted an umbrella review which is a comprehensive and systematic approach that collects and critically evaluates all systematic reviews and meta-analyses performed on a specific research topic [10]. We used previously described, standardized methods that have been already used in published umbrella reviews referring to risk factors related to various outcomes [11–14] and have been elaborated below.

A protocol for this umbrella review was registered in the International prospective register of systematic reviews (PROSPERO 2021 CRD42021227296).

### Search strategy

Two researchers (A.E., I.M.) independently searched PubMed and Scopus databases from inception to February 2021, to identify systematic reviews with meta-analyses of studies that examine the association between risk factors and preterm birth. The search strategy included combinations of the Medical Subject Headings (MESH) terms, keywords, and word variants for terms “preterm birth” AND (“systematic review” OR “meta-analysis”). Titles and abstracts were screened, and potentially eligible articles were retrieved for full-text evaluation. A detailed description of our search strategy is provided in Additional file 1.

### Eligibility criteria and data extraction

We included systematic reviews with meta-analyses investigating the association between various types of exposures as risk factors for PTB. Specifically, we included studies with singleton pregnancies and studies where PTB was evaluated as the primary outcome. Case reports or series and individual participant data meta-analyses were excluded. We also excluded studies that set time limits on time span or were performed on a restricted setting (i.e., conducted for one specific country). Studies that assessed PTB as a secondary outcome were also excluded. We excluded meta-analyses that assessed PTB as a secondary outcome for two reasons; first, in any analysis of a secondary outcome, there is a possibility of lack of power to detect an effect, given that studies design their power calculations based on a primary outcome. Therefore, any assessment of effect size, heterogeneity, and other statistics would be meaningless under the lack of power. Second, some components of the grading of evidence, such as publication bias, are assessed based on the primary outcome of the studies and could not be evaluated for secondary outcomes. Furthermore, we excluded studies including multiple pregnancies and studies that assessed genetic or over -omics features as risk factor for PTB. All studies were compared to avoid the possibility of duplicate or overlapping samples. If more than one meta-analysis referring to the same research question was eligible, parameters such as the largest amount of component studies with data on individual studies’ effect sizes, publication year, and in some cases the number of participants on individual studies were considered to retain the appropriate one for the main analysis.

Publications whom estimates of the studied associations, such as relative risks (RR) and 95% confidence intervals (CIs) were not reported or could not be retrieved/calculated were excluded from the analysis. For the non-environmental risk factors, we also excluded meta-analyses that did not provide the number of cases in the exposed and non-exposed groups, which is used for the calculation of the excess significance test. For the environmental risk factors, since most commonly they report the results as per unit(s) increase in exposure and the entire population is exposed, we included them even if they did not report the number of cases and total sample size. As most of the included meta-analyses did not report the number of cases or the sample size of the studies included, we were unable to estimate the power of each meta-analysis and the excess significance test.

Eligible articles were screened by four independent reviewers (AE/IM and EB/TK). Any disagreement between reviewers was resolved by consensus or after the evaluation of a third author (SP or EE). The data of eligible studies were extracted in a predefined data extraction form recording for each study the first author, journal, year of publication, the examined risk factors, and the number of reviewed studies. Either the study-specific relative risk estimates (risk ratio, odds ratio, hazard ratio, incidence rate ratio) and the confidence intervals were extracted or the mean and the standard deviation for continuous outcomes were also noted in this form. We also extracted the exposed and control groups used, outcome assessed, study population, exposure characteristics, number of studies in the meta-analysis, meta-analysis metric and method, effect estimate with the corresponding 95% confidence interval, number of cases and total sample size, *I*^2^ metric and the corresponding *χ*^2^ *P*-value for the *Q* test, and Egger’s regression *P*-value.

### Assessment of summary effect and heterogeneity

We re-calculated summary effects and 95% confidence intervals (CIs) for each meta-analysis via fixed and random effects model [15, 16]. 95% prediction intervals (PI) were also computed for the summary random-effects estimates, which further account for between-study heterogeneity indicating the uncertainty for the effect that would be expected in a new study examining the same association [17, 18]. A PI describes the variability of the individual study estimates around the summary effect size and represents the range in which the effect estimate of a new study is expected to lie.

The largest study was considered to be the most precise if there was a difference between the point estimate and the upper or lower 95% confidence interval less than 0.20 [19]. If the largest study presented a statistically significant effect, then we recorded this as a part of the grading criteria.

Between-study heterogeneity was assessed and *P*-value of the *χ*^2^-based Cochran Q test and the *I*^2^ metric for inconsistency (reflecting either diversity or bias) was reported, too. *I*^2^ metric was used to indicate the ratio of between-study variance over the sum of within- and between-study variances, ranging from 0 to 100% [20]. Values exceeding 50% or 75% are usually considered to represent large or very large heterogeneity, respectively [21]. 95% confidence intervals were calculated as per Ioannidis et al. [21].

### Assessment of small-study effects

Small studies tend to give substantially larger estimates of effect size when compared to larger studies. We evaluated the evidence of the presence of the small-study effects, to identify publication and other selective reporting biases. They can also reflect genuine heterogeneity, chance, or other reasons for differences between small and large studies [22]. We evaluated whether smaller (less precise) studies lead to inflated effect estimates compared to larger studies. We used the regression asymmetry test proposed by Egger, which examines the potential existence of small-study effects via funnel plot asymmetry [23]. Egger’s test fits a linear regression of the study estimates on their standard errors weighted by their inverse variance. Indication of small-study effects based on Egger’s asymmetry test was claimed when *P*-value ≤ 0.10. This is considered as an indication of publication bias; indication of small-study effects based on Egger’s asymmetry test was claimed when *P*-value ≤ 0.10 and the random effects summary estimate was larger compared to the point estimate of the largest (most precise) study in the meta-analysis.

### Excess statistical significance evaluation

The excess significant test was applied to evaluate the existence of a relative excess of significant findings in the published literature for any reason (e.g., publication bias, selective reporting of outcomes or analyses) [24]. This is a binomial test evaluating whether the number of positive studies in a meta-analysis was too large according to the power that these studies have to detect plausible effects at *α* = 0.05. The power of each component study was calculated using the fixed-effects summary, the random effects summary, or the effect size of the largest study (smallest SE) as the plausible effect size [13] using an algorithm using non-central *t* distribution to calculate the power of each study [25]. Excess statistical significance for single meta-analyses was claimed at *P* < 0.10 (one-sided *P* < 0.05, with observed > expected as previously proposed), given the power to detect a specific excess will be low, especially with few positive studies [24].

### Grading of evidence

We followed a 4-level grading (robust, highly suggestive, suggestive, and weak) to evaluate the strength of the evidence based on the following criteria: number of cases, summary random-effects *P*-value, between-studies heterogeneity, 95% PI, small-study effects bias, and excess statistical significance [26]. This grading approach based on these parameters was used because it allows for an objective, standardized classification of the level of evidence and has been previously shown to provide consistent results with other more subjective grading schemes [26–30].

Briefly, meta-analyses were considered to be supported by robust evidence if the association was supported by more than 1000 cases, a highly significant association (the random effects model had a *P*-value ≤ 10^−6^, a threshold that is considered to substantially reduce false positive findings) [31–33], there was absence of high heterogeneity based on *I*^2^ < 50%, the 95% PI excluded the null value, and there was no evidence of small-study effects or excess statistical significance. Highly suggestive evidence required more than 1000 cases, a highly significant association (a random-effects *P*-value ≤ 10^−6^), and the largest study in the meta-analysis was significant at *P* < 0.05. Associations based on meta-analyses with a random-effects *P*-value ≤ 10^−3^ and included more than 1000 cases [31–33] were graded as suggestive evidence. The remaining significant associations at *P* < 0.05 were graded as weak evidence. We need to highlight that this specific grading scheme focuses on the reduction of false-positive findings and the evaluation of potential biases in the studied associations. Therefore, the set of criteria used here is not ideal for a detailed evaluation of non-significant associations and to distinguish insufficient evidence from robust evidence of no association. That would require a different approach and another set of criteria altogether that would focus on the power of the meta-analyses to observe a significant effect, which was beyond the scope of our review. This grading system has been evaluated [34] and showed that these criteria may offer relatively independent and complimentary insights into the evidence of an observational association. Other systems such as GRADE [35] and ROBIS [36] have focused mainly on evaluating randomized evidence from RCTs or non-randomized studies of intervention.

Statistical analyses were performed using STATA version 14 (StataCorp, TX, USA).

## Results

### Description of eligible meta-analyses

**Fig. 1:**
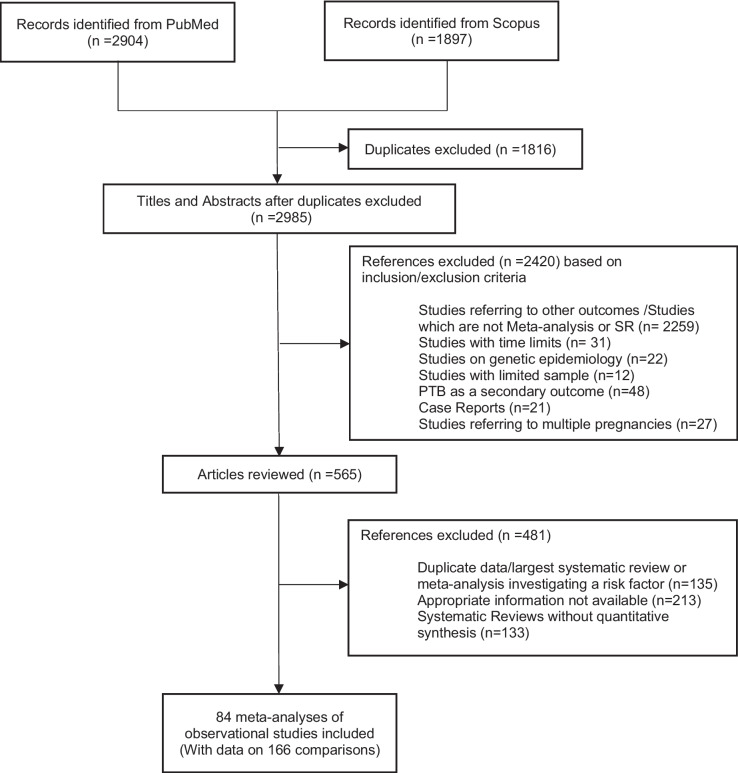
Flow diagram for the selection of included studies

The search identified 2985 items, of which 2420 were excluded after a review of the title and abstract (Fig. 1, PRISMA flowchart). Of the remaining 565 articles that were reviewed in full text, eight articles did not report the appropriate information for the calculation of excess of statistical significance (either because the total sample size was missing or the study-specific relative risk estimates were missing), and 134 articles were excluded because a larger systematic review or meta-analysis investigating the same risk factor was available. From the 219 comparisons, we further excluded the ones that included one or two studies (53 comparisons). Therefore, 218 articles were analyzed, of which 133 were systematic reviews without any quantitative component and 84 were meta-analyses. The 84 eligible meta-analyses [4, 6–9, 37–115] included data on 166 comparisons and 1480 primary studies.

### Summary effect sizes and significant findings

Three to 152 studies, with a median of 11 studies, were included per meta-analysis. The median number of cases and total population in each study were 91 and 1004, respectively. The median number of cases and total population in each meta-analysis was 7266 and 94,907, respectively. The number of cases was greater than 1000 in 94 comparisons. Overall, 570 (38,5%) individual studies observed statistically significant results at *P* < 0.05. Thirty-nine meta-analyses used the Newcastle-Ottawa scale to assess qualitatively the included primary studies. One meta-analysis used assessment criteria for non-randomized observational studies adapted from Duckitt and Harrington, 3 meta-analyses used the Methodological Index for Non-Randomized Studies (MINORS), and 37 meta-analyses used other assessment tools. Four meta-analyses did not perform any quality assessment. Details of the 166 comparisons that included 1480 individual study estimates are summarized in Additional file 2.

Of the 166 comparisons, 99 (59.3%) had statistically significant findings at *P* < 0.05 using the random-effects model, of which 93 reported an increased risk and six a decreased risk for preterm birth. The associations identified to decrease the risk of PTB are the following: preconception care vs no care [47], magnesium supplementation vs placebo [72], single vs double embryo transfer [87], high gestational weight gain vs normal gestational weight gain [106], interpregnancy interval following miscarriage < 6 months vs > 6 months [108], and greenery including only a 100-m normalized difference vegetation index (NDVI) buffer [113]. Of these, a total of 61 (36.8%) associations presented statistically significant effect at *P* < 0.001, while only 41 (24.7%) remained significant after the application of a more stringent *P*-value threshold of *P* < 10^−6^ (Table 1).

**Table 1:** Assessment across the statistically significant associations for preterm birth

Level of evidence	Criteria
**Robust**	> 1000 cases, ^a^ *P* < 10^−6^, not large heterogeneity (*I*^2^ < 50%), 95% prediction interval excluding the null value, no evidence for small-study effects^b^, and excess significance bias ^c^
Risk factors supported by robust evidence	Amphetamines [53]
	Isolated single umbilical artery [60]
	Maternal personality disorder [70]
	SDB (objective assessment) [80]
	Prior I-TOP with VA [98]
	Low GWG [107]
	IPI following miscarriage of < 6 m (compared to IPI following miscarriage of ≥ 6 months, with Conde-Agudelo A, 2004 excluded) [108]
**Highly Suggestive**	> 1000 cases, ^a^ *P* < 10^−6^ and statistically significant effect present at the largest study at *P* < 0.05
Risk factors supported by highly suggestive evidence	1^st^ trimester bleeding [51]
	Prior surgical I-TOP (for PTB in singleton pregnancies) [98]
	Obstetric cholestasis [66]
	PCOS [115]
	Cancer survivors [41]
	Placenta previa [43]
	African/Black race [49]
	Aboriginal ethnicity [50]
	BMI of > 40 kg/m^2^ (compared to BMI = 30–34.9 kg/m^2^) [75]
	BMI of > 40 kg/m^2^ (compared to BMI = 30–39.9 kg/m^2^) [75]
	Endometriosis (combined spontaneous conception and assisted reproduction) [8]
	Endometriosis (spontaneous conception) [8]
	Maternal age of ≥ 45 years old [103]
	CKD during pregnancy [104]
	Underweight women [105]
	Maternal vitamin D status (for spontaneous PTB) [110]
	SMM: hemorrhagic disorders [68]
	SMM: hepatic disorders [68]
	LEEP [111]
	LLETZ for CIN [112]
	Any type of treatment for CIN with a cone depth of ≥ 10–12 mm (compared to untreated CIN) [112]
	Any type of treatment for CIN with a cone depth of ≥ 15–17 mm (compared to untreated CIN) [112]
	Intimate partner violence [39]
	Unmarried women [52]
	Cocaine [79]
	Entire pregnancy high level PM_2.5_ exposure [48]
**Suggestive**	> 1000 cases, ^a^ *P* < 10^−3^
Risk factors supported by suggestive evidence	Pre-gravid OC use [40]
	Marijuana during pregnancy [57]
	SMM: thromboembolic disorders [68]
	Periodontal disease [74]
	Women of short stature [77]
	Antipsychotics during pregnancy [38]
	*Trichomonas vaginalis* infection [84]
	Blastocyst-stage embryo transfer (vs cleavage embryo transfer) [86]
	Fresh blastocyst transfer (for PTB) [89]
	Fresh blastocyst transfer (for very PTB < 32 weeks) [89]
	HPV Infection (crude) [7]
	HPV Infection (age adjusted) [7]
	> 1 prior surgical I-TOP [98]
	Any type of treatment for CIN with a cone depth of ≥ 20 mm (compared to untreated CIN) [112]
	Greenery (including only a 100-m NDVI buffer) [113]
**Weak**	The rest associations with^a^*P* < 0.05
Risk factors supported by weak evidence	History of preterm twins [4]
	History of preterm twins 34–36 + 6 weeks [4]
	History of preterm twins 30–33 + 6 weeks [4]
	History of preterm twins < 30 weeks [4]
	History of spontaneous twin preterm birth [4]
	History of spontaneous twin preterm birth 34–36 + 6 weeks [4]
	Subseptate uterus [46]
	Cancer survivors treated after radiotherapy [41]
	H1 Antihistamine [37]
	Velamentous cord insertion [42]
	Metformin [44]
	Diabetic nephropathy in T1DM [45]
	Preconception care [47]
	Asian race [49]
	Hispanic ethnicity [49]
	Laparoscopic appendectomy [9]
	Hyperemesis gravidarum (cohort studies) [54]
	Hyperemesis gravidarum (case control studies) [54]
	Arcuate uterus [46]
	Septate uterus [46]
	Bicornuate uterus [46]
	Didelphys uterus [46]
	Unicornuate uterus [46]
	Triptan [55]
	Migraine [55]
	Topical retinoids (exposed infants) [56]
	Hydroxychloroquine [56]
	TB [58]
	Multivitamins [59]
	Fetus with small thymus [61]
	Probiotics during pregnancy (for PTB < 34 weeks) [62]
	Probiotics during pregnancy (for PTB < 37 weeks) [62]
	Home visits for pregnant women [63]
	APS [64]
	Bed Rest (in developing regions, for PTB < 37 weeks) [65]
	Bed Rest (in developed regions, for PTB < 37 weeks) [65]
	Bed Rest (in developing regions, for very PTB) [65]
	Bed Rest (in developed regions, for very PTB) [65]
	Pregnancy-associated malaria [67]
	Nicotine Replacement Therapy [69]
	Women involved in motor vehicle crashes [71]
	Magnesium supplementation [72]
	Donor sperm (for PTB) [73]
	Donor sperm (for very PTB) [73]
	Bariatric surgery [76]
	Vitamin C and others supplementation [78]
	SDB (questionnaire-based assessment) [80]
	Asthma with exacerbation during pregnancy [81]
	Asthma without exacerbation during pregnancy [81]
	Alcohol consumption before or during pregnancy [83]
	Vaginal clindamycin treatment for bacterial vaginosis [85]
	Single embryo transfer (randomized clinical trials) [87]
	Single embryo transfer (cohort studies) [87]
	Stimulated cycle IVF [88]
	Bacterial vaginosis [90]
	Intermediate vaginal flora [90]
	HPV 6/11/16/18 vaccine in periconceptional period or during pregnancy [6]
	Quinolones during 1st trimester [91]
	Macrolides [92]
	Clindamycin [92]
	Metronidazole alone or in combination [92]
	Metronidazole [92]
	Dental caries [93]
	Celiac disease [94]
	Single-twin death after 14 weeks of monochorionic pregnancy [95]
	Prenatal care (observational studies) [96]
	Prenatal care (randomized clinical trials) [96]
	Endometriosis (assisted reproduction) [8]
	Knowledge of TVU-measured CL in singletons pregnancies with symptoms of PTL [97]
	Only 1 prior surgical I-TOP [98]
	Prior 1st trimester surgical I-TOP [98]
	Prior S-TOP [98]
	Prior uterine evacuation [98]
	Prior I-TOP [98]
	Prior I-TOP with dilation and evacuation [98]
	Hyperthyroidism [99]
	Clinical hypothyroidism [100]
	Subclinical hypothyroidism [100]
	Hypothyroxinemia [100]
	LT4 treatment in euthyroid women with thyroid autoimmunity (with Negro R, 2016 included) [101]
	LT4 treatment in euthyroid women with thyroid autoimmunity (with Negro R, 2016 excluded) [101]
	Primiparous mother [102]
	High GWG [106]
	IPI following miscarriage of < 6 months (compared to IPI following miscarriage of ≥ 6 months,
	with Conde-Agudelo A, 2004 included) [108]
	IPI following miscarriage < 6 months (compared to IPI following miscarriage of 6–12 months) [108]
	IPI following miscarriage < 6 months (compared to IPI following miscarriage of > 12 months) [108]
	Treated CIN (for PTB < 37 weeks) [109]
	Treated CIN during pregnancy [109]
	Treated CIN before pregnancy [109]
	Untreated CIN [109]
	Treated CIN (for spontaneous PTB < 37 weeks) [109]
	Treated CIN (for PTB < 32 weeks) [109]
	maternal 25-OHD concentration of < 50 nmol/L [110]
	maternal 25-OHD concentration of < 75 nmol/L [110]
	Vitamin D supplementation [110]
	Maternal Vitamin D status (for PTB in general) [110]
	Any type of treatment for CIN with a cone depth of ≤ 10–12 mm (compared to untreated CIN) [112]
	Any type of treatment for CIN with a cone depth of ≥ 10–12 mm (compared to any type of treatment for CIN with a cone depth of ≤ 10–12 mm) [112]
	Any type of treatment for CIN with a cone depth of ≥ 15–17 mm (compared to any type of treatment for CIN with a cone depth of ≤ 15–17 mm) [112]
	Any type of treatment for CIN with a cone depth of ≥ 20 mm (compared to any type of treatment for CIN with a cone depth of ≤ 20 mm) [112]
	1st trimester PM_2.5_ exposure [48]
	Entire pregnancy PM_2.5_ exposure [48]
	1st trimester high-level PM_2.5_ exposure [48]
	1st trimester low level PM_2.5_ exposure [48]
	Entire pregnancy low level PM_2.5_ exposure [48]
	Entire pregnancy PM_2.5_ exposure [114]
	1^st^ trimester PM_2.5_ exposure [114]
	2^nd^ trimester PM_2.5_ exposure [114]
	3^rd^ trimester PM_2.5_ exposure [114]
	1^st^ month PM_2.5_ exposure [114]
	Within 1 month before birth PM_2.5_ exposure [114]
	Individual-level PM_2.5_ exposure [114]
	Semi-individual-level PM_2.5_ exposure [114]
	Regional-level PM_2.5_ [114]
	PM_2.5_ exposure [114]
	1^st^ trimester NO_2_ exposure [82]
	2^nd^ trimester NO_2_ exposure [82]
	3^rd^ trimester NO_2_ exposure [82]
	Whole pregnancy NO_2_ exposure [82]

*Abbreviations: I-TOP* Induced termination of pregnancy, *S-TOP*, Spontaneous termination of pregnancy, *TOP* Termination of pregnancy, *PCOS* Polycystic ovary syndrome, *APS* Antiphospholipid syndrome, *GWG* Gestational weight gain, *T1DM* Type 1 diabetes mellitus, *SDB* Sleep-disordered breathing, *SMM* Severe maternal morbidity, *BMI* Body mass index, *NRT* Nicotine replacement therapy, *TVU* Transvaginal ultrasound, *CL* Cervical length, *PTL* Preterm labor, *CKD* Chronic kidney disease, *IPI* Interpregnancy interval, *CIN* Cervical intraepithelial neoplasia, *25-OHD* 25-hydroxyvitamin D, *LEEP* Loop electrosurgical excision procedure, *LLETZ* Large loop excision of transformation zone, *OC* Oral contraceptive, *LT4* Levothyroxine, *HPV* Human papillomavirus, *TB* Tuberculosis, *IVF* In vitro fertilization, *PM*_*2.5*_ Particulate matter with aerodynamic diameter less than or equal to 2.5 μm; *PM*_*10*_ Particulate matter with aerodynamic diameter less than or equal to 10 μm, *NO*_*2*_ Nitrogen dioxide

^a^*P* indicates the *P*-values of the meta-analysis random effects model

^b^Small-study effect is based on the *P*-value from Egger’s regression asymmetry test (*P* < 0.10)

^c^Based on the *P*-value (*P* < 0.05) of the excess significance test using the largest study (smallest standard error) in a meta-analysis as the plausible effect size

### Between-study heterogeneity and prediction intervals

Forty-four (26.5%) comparisons had large (*I*^2^ ≥ 50% and ≤ 75%) and 47 comparisons (28.3%) had very large (*I*^2^ > 75%) heterogeneity estimates (see Additional file 2). When calculating the 95% PIs, the null value was excluded in only thirty-one (18.7%) comparisons.

### Small-study effects

Evidence for statistically significant small-study effects (Egger test *P* < 0.10 and random-effects summary estimate larger compared with the point estimate of the largest study in the meta-analysis) was identified in 37 (22.3%) comparisons (see Additional file 2).

### Test of excess statistical significance

Evidence of excess-statistical-significance bias was observed in 12 (7.2%) associations, with statistically significant (*P* < 0.05) excess of positive studies under any of the three assumptions for the plausible effect size, i.e., the fixed-effects summary, random-effects summary or results of the largest study (see Additional file 2). In addition, the observed and expected number of positive studies showed that, overall, the excess of positive results was driven by meta-analyses with large estimates of heterogeneity (*I*^2^ > 50%).

### Grading of evidence

The summary of the epidemiological credibility for 166 associations of risk factors for PTB is shown in Additional file 2. Seven of the 166 associations (4.2%) were supported by robust evidence (amphetamines, fetus with isolated single umbilical artery, maternal personality disorder, sleep-disordered breathing (SDB), prior induced termination of pregnancy (I-TOP) with vacuum aspiration (VA), low gestational weight gain, and interpregnancy interval (IPI) following miscarriage less than 6 months) (see Additional file 3). Twenty-six associations (15.7%) were supported by highly suggestive evidence referring to obstetric history, medical history, social and economic profile, and drugs (see Table 1). Fifteen associations (9%) were supported by suggestive evidence, including pre-gravid oral contraceptive use, marijuana, severe maternal morbidity (SMM), periodontal disease, women of short stature, antipsychotics during pregnancy, *Trichomonas vaginalis* infection, blastocyst-stage embryo transfer (vs cleavage stage embryo-transfer), fresh blastocyst transfer (for PTB), fresh blastocyst transfer (for very PTB), HPV infection (crude), HPV infection (age-adjusted), > 1 prior surgical I-TOP, any type of treatment for cervical intraepithelial neoplasia (CIN) with a cone depth ≥ 20 mm (compared to untreated CIN), and greenery.

Regarding the environmental risk factors, higher residential greenness did not technically qualify to be categorized as robust evidence because the random effects *P*-value was 3.25 × 10^−6^ but fulfilled all other criteria. The rest of the associations regarding different levels of exposure to air pollutants [particulate matter with aerodynamic diameter less than or equal to 2.5 μm (PM_2.5_), nitrogen dioxide (NO_2_)] in all windows of exposure were classified as weak.

## Discussion

In this umbrella review, we evaluated the current evidence, derived from meta-analyses of observational studies on the association between various risk factors and PTB. Overall, from the 166 associations that have been examined, only 4.2% had epidemiologically robust results with no suggestion of bias, as can be inferred by substantial heterogeneity between studies, small-study effects, and excess significance bias. Seven risk factors were supported by robust evidence, including amphetamine exposure [53], isolated single umbilical artery [60], maternal personality disorder [70], sleep-disordered breathing measured with objective assessment [80], prior induced termination of pregnancy with vacuum aspiration compared to no termination [98], low gestational weight gain compared to normal weight gain [107], and interpregnancy interval following miscarriage less than 6 months compared to more than 6 months [108]. Several others had highly suggestive evidence including intimate partner violence [39] and unmarried women [52], cancer survivors [41], African/Black race [49], placental previa [43], hemorrhagic and hepatic disorders [68], endometriosis [8], chronic kidney disease [104], and treatments for CIN [112].

### Interpretation in the light of evidence

Some of the risk factors identified from our analysis as robust are well-known risk factors and have been incorporated into the screening processes during prenatal visits such as illicit drug use, ultrasonographic markers, and reproductive history [5]. Nevertheless, we identified a few that are not receiving the attention they should during prenatal visits even though they demonstrate robust evidence.

This includes maternal psychosocial profile and sleeping quality that are either rarely screened during prenatal visits or not considered by clinicians as risk factors for PTB. Traditionally, emphasis was given to factors such as cervix length and history of PTB and their obstetric management [5]. Screening and early intervention on maternal personality disorders and SDB during pregnancy should be further evaluated at prenatal visits and potentially contribute to PTB prevention.

### Interpregnancy intervals

One association with highly controversial evidence is interpregnancy interval following a miscarriage and the risk of preterm birth. The World Health Organization (WHO) encourages women who experienced a previous miscarriage to wait for a minimum of 6 months before the next conception to achieve optimal outcome and reduce obstetric complications such as preterm birth [116]. Contrary to the findings of the research on which WHO based its recommendations, some studies reported that the risk of adverse obstetric outcomes including preterm birth is lower in women who conceived less than 6 months after a pregnancy loss [117–119], while synthesizing all available data provided the same conclusion [108]. This meta-analysis included eight studies and performed two analyses: one including the study of Conde Agudelo 2004 [120] and one excluding it, and robust results were obtained after excluding this study. While this was a large retrospective study on which the WHO guidelines for delaying pregnancy for at least 6 months [116] are based, it did not differentiate between induced and spontaneous abortions and used data from many countries where induced abortion is illegal [120], therefore should be interpreted with caution. More recent studies have criticized methods used in the previous studies; therefore, the question remains open as to the causal effect of short interpregnancy intervals after miscarriage on adverse obstetric outcomes remains unknown [121, 122]. After a miscarriage, there is a very small burden on the folate reserve, and thus, miscarriage is not very likely to lead to folate deficiency in the postpartum period, so miscarriage and delivery later in pregnancy may have differential effects on subsequent pregnancy [123, 124]. This could explain the reduced risk of adverse outcomes in a short IPI after a miscarriage [123] but not after delivery. In support of this hypothesis, there is evidence to suggest that late miscarriages (after 12 weeks of gestation) are associated with worse outcomes in the subsequent pregnancy [124]. In addition, most women who attempt another pregnancy soon after a miscarriage are likely to be motivated to take better care of their health and consequently result in better pregnancy outcomes [125–127]. Another plausible reason may be that those who conceive soon after a miscarriage are naturally more fertile and younger and consequently have better pregnancy outcomes. Therefore, even though the characteristics of the meta-analysis included in our assessment classified this association as robust for a protective effect, given the complex causal structure of these associations, interpretations should be made with caution.

### Sleep disorders and mental health

Another risk factor with robust evidence was sleep-disordered breathing. This meta-analysis clearly demonstrated the increased risk profile of women who experience SDB not only for preterm birth but for other adverse pregnancy outcomes [80]. Regarding plausible mechanisms, the association between SDB and intermittent maternal hypoxia as well as the link with conditions synonymous with impaired placental function such as pre-eclampsia suggests a multifactorial cause, with both physiologic changes associated with pregnancy and placental dysfunction involved [80]. This robust association has clear implications for obstetric practice. First, given the rapidly increasing worldwide obesity rates, SDB is likely to become more prevalent in the pregnant population and it should be introduced in screening. Second, the increased risk for both adverse intrapartum and perinatal outcomes demonstrated in this review strongly supports the need for increased surveillance in women who experience SDB during pregnancy. Third, public health education programs must take into account the specific maternal and perinatal risks and promote education about the significance of obstructive sleep apnea symptoms and the need for women to discuss this with their obstetric caregivers. Screening for sleeping habits and suggesting more frequent follow-up for women with such symptoms have the potential to reduce the burden of PTB.

In alignment with this suggestion, women with personality disorders could be identified early through mental health screening, where targeted health interventions and multidisciplinary management can be implemented to reduce adverse outcomes for the baby/child and woman. This early identification and support also have the potential to enable the prevention of maladaptive development trajectories within the mother-infant relationship [128, 129].

The ability to modify those factors, mainly those related to mental health and sleep quality screening, through clinical interventions or public health policy measures remains to be established. Nevertheless, we need to highlight that there is no guarantee that even a convincing observational association for a modifiable risk factor would necessarily translate into large preventive benefits for preterm birth if these risk factors were to be modified [8].

### Clinical practice and medical history

Another association that fulfilled all criteria for a robust association is the prior I-TOP with VA. Concerns have been expressed regarding the validity of the reported association mainly due to the quality of the primary studies [130]. Many of them did not adjust for strong confounders such as parity, prior PTB, race, and smoking [98, 130]. The analysis of primary studies that reported data on cofounders and adjusted the risk estimated on these cofounders, revealed a greater increase in the PTB incidence [98]. This is supported by the fact that women who underwent an I-TOP usually have a low socioeconomic status and are likely to be exposed to a variety of factors related to PTB [98]. Moreover, abortion is a reported outcome that is accompanied by social stigma and, therefore, can be omitted from the medical history, leading to a high risk of differential misclassification. This highlights the need to thoroughly examine the other possible biases that can be identified in a meta-analysis even in the case that the epidemiologic criteria classify an association as robust.

Furthermore, it is important that clinical examination and medical history includes risk factors which are not well known, identified in meta-analysis with highly suggestive evidence. Regarding highly suggestive evidence, there were a few that are well known and used to classify pregnancies as high risk for PTB such as therapies for cervical intraepithelial neoplasia, advanced maternal age, placental pathology, race, first trimester bleeding, and maternal comorbidities. There were also included factors that are not routinely screened in the obstetric population such as intimate partner violence, cancer survivors, and being unmarried.

Obesity is generating an unfavorable metabolic environment from early gestation; therefore, initiation of interventions for weight loss during pregnancy might be belated to prevent or reverse adverse effects, which highlights the need for weight management strategies before conception [75, 106, 107, 131]. Moreover, obesity is becoming a global epidemic, while assessing the strength of evidence that supports the impact of overweight and obesity in comorbidities such as sleep-disordered breathing could allow not only the identification of women at high risk for adverse outcomes including PTB, but also better prevention. PTB does not only increase the risk for maternal and infant complications, but also significantly increases a woman’s risk of cardiovascular disease (CVD) after pregnancy; therefore, primary prevention of obesity could lead to multiple benefits [132–135].

### Environment

Regarding environmental risk factors, increased residential greenness was associated with a protective effect on the risk of PTB. Although this finding was categorized as having suggestive evidence, the *P*-value of the random effect estimate was very close to the stringent threshold of < 10^−6^. Acknowledging the detrimental projected effect of climate change in greenness and given that it is one of the few protective risk factors for PTB [113], serious efforts should be made to maintain and grow residential greenness. Possible mechanisms include among others amelioration of the effects of air pollutants, reduction of stress, and increase in physical activity [113]. There was also suggestive evidence for early pregnancy exposure to PM_2.5_ and the risk of PTB. This association has been debated in the literature with conflicting results about the timing and magnitude of effect and is less robust than other associations that have been shown to have strong evidence for associations [136] such as birth weight.

### Strengths and limitations

To our knowledge, this umbrella review represents the most comprehensive overview of published literature of observational studies to date investigating associations between a wide array of risk factors and PTB. The epidemiological robustness of meta-analyses of observational studies was assessed against a transparent and replicable set of statistical criteria. In addition, we performed a deeper assessment of these associations and assessed their potential to test causal assumptions. Our assessment has certain limitations. Umbrella reviews focus on existing systematic reviews and meta-analyses and therefore some studies may have not been included either because the original systematic reviews did not identify them, they were too recent to be included, or they did not provide the data to be included. In the current assessment, we used all available data from observational studies; therefore, the meta-analysis estimates may partly reflect the biases in the primary studies. Statistical tests of bias in the body of evidence (small-study effect and excess significance tests) offer hints of bias, not definitive proof thereof, while the Egger test is difficult to interpret when the between-study heterogeneity is large. These tests have low power if the meta-analyses include less than 10 studies and they may not identify the exact source of bias [22, 24, 34]. More specifically, in our study, all robust evidence applied to meta-analyses with less than 10 studies; therefore, the results of publication bias should be interpreted with caution. Furthermore, we did not appraise the quality of the individual studies on our own, since this should be included in the original meta-analysis and it was beyond the scope of the current umbrella review. However, we recorded whether and how they performed a quality assessment of the synthesized studies. Lastly, we cannot exclude the possibility of selective reporting for some associations in several studies. For example, perhaps some risk factors were more likely to be reported, if they had statistically significant results. Diving deeper into the associations that were classified as robust, we detected some issues beyond the prespecified criteria that are traditionally applied for umbrella reviews.

Therefore, it is recommended that future umbrella reviews perform a comprehensive assessment of the associations beyond the classic criteria.

## Conclusions

The present umbrella review of meta-analyses identified 166 unique risk factors for preterm birth. Our analysis identified seven risk factors with robust evidence and strong epidemiological credibility pertaining to isolated single umbilical artery, amphetamine exposure, maternal personality disorder, sleep-disordered breathing, induced termination of pregnancy with vacuum aspiration, low gestational weight gain, and interpregnancy interval following miscarriage of less than 6 months, but the results should be interpreted with caution. As previously suggested, the use of standardized definitions and protocols for exposures, outcomes, and statistical analyses may diminish the threat of biases, enhance comparability of different studies examining risk factors, and promote the development and training of prediction models that could identify high-risk populations and promote public health.

### Supplementary Information

**Additional file 1. **Search Strategy for Umbrella Review.

**Additional file 2. **Details of the comparisons.

**Additional file 3. **Details of the comparisons with robust evidence.

**Additional file 4. **Details of the protective factors.

## Data Availability

Relevant data to our study are mainly included in the article, tables, and supplemental material. However, we will share the original dataset after reasonable requests.

## Abbreviations

25-OHD: 25-Hydroxyvitamin D
APS: Antiphospholipid syndrome
BMI: Body mass index
CIN: Cervical intraepithelial neoplasia
CIs: Confidence intervals
CKD: Chronic kidney disease
CL: Cervical length
CVD: Cardiovascular disease
GWG: Gestational weight gain
HPV: Human papillomavirus
IPI: Interpregnancy interval
I-TOP: Induced termination of pregnancy
IVF: In vitro fertilization
LEEP: Loop electrosurgical excision procedure
LLETZ: Large loop excision of transformation zone
LT4: Levothyroxine
MESH: Medical Subject Headings
MINORS: Methodological index for non-randomized studies
NO_2_: Nitrogen dioxide
NRT: Nicotine replacement therapy
NVDI: Normalized difference vegetation index
OC: Oral contraceptive
PCOS: Polycystic ovary syndrome
PI: Prediction interval
PM_10_: Particulate matter with aerodynamic diameter less than or equal to 10 μm
PM_2.5_: Particulate matter with aerodynamic diameter less than or equal to 2.5 μm
PTB: Preterm birth
PTL: Preterm labor
RR: Relative risk
SDB: Sleep-disordered breathing
SMM: Severe maternal morbidity
S-TOP: Spontaneous termination of pregnancy
T1DM: Type 1 diabetes mellitus
TB: Tuberculosis
TOP: Termination of pregnancy
TVU: Transvaginal ultrasound
WHO: World Health Organization
