# Supplementary material for: Risk factors for preterm birth: an umbrella review of meta-analyses of observational studies
Source: BMC Med. 2023 Dec 13;21:494. doi: 10.1186/s12916-023-03171-4 (PMC10720103; doi:10.1186/s12916-023-03171-4)
Supplement: Supplementary file 1 — Additional file 1. Search Strategy for Umbrella Review. [file 12916_2023_3171_MOESM1_ESM.docx]

**Additional file 1:**

**Search Strategy for Umbrella Review**

**Database:** PubMed

**1.Time Period:**

From Inception to 28/02/2021

**2. Search Strategy:**

| **Search Strategy** |
| --- |
| ("preterm birth" OR "preterm delivery" OR "preterm labor" OR "prematurity" OR "premature birth [MESH]" OR "premature birth" OR "premature delivery" OR "premature labor") AND ("systematic review" OR "meta-analysis") |

**Database:** Scopus

**1.Time Period:**

From Inception to 28/02/2021

**2.Search Strategy:**

| **Search Strategy** |
| --- |
| ( TITLE ( "preterm birth" OR "preterm delivery" OR "preterm labor" OR "prematurity" OR "premature birth [mesh]" OR "premature birth" OR "premature delivery" OR "premature labor" AND "systematic review" OR "meta-analysis" ) OR ABS ( "preterm birth" OR "preterm delivery" OR "preterm labor" OR "prematurity" OR "premature birth [mesh]" OR "premature birth" OR "premature delivery" OR "premature labor" AND "systematic review" OR "meta-analysis" ) ) AND PUBYEAR > 1986 AND PUBYEAR < 2022 AND ( LIMIT-TO (SUBJAREA , "medi" ) ) AND ( LIMIT-TO ( LANGUAGE , "english" ) ) |
