# Supplementary material for: Risk factors for preterm birth: an umbrella review of meta-analyses of observational studies
Source: BMC Med. 2023 Dec 13;21:494. doi: 10.1186/s12916-023-03171-4 (PMC10720103; doi:10.1186/s12916-023-03171-4)
Supplement: Supplementary file 2 — Additional file 2. Details of the comparisons. [file 12916_2023_3171_MOESM2_ESM.docx]

| Level of Evidence | Area | Author, year | Comparison | Studies | Cases/controls | Random effects* | Fixed effects**†** | Largest effect**‡** | Egger§ | I2(95%CI)(P)**\|\|** | 95% PI**≠** | O**¶** | P**  (fixed) | P**  (random) | E  (largest)ȣ | P**  (largest) |
| --- | --- | --- | --- | --- | --- | --- | --- | --- | --- | --- | --- | --- | --- | --- | --- | --- |
| Robust | Drugs | Ladhani NN, 2011 | Amphetamines | 5 | 641/61429 | 4.11 | 4.56 | 5.24 | 0.12 | 0.38(0-0.76)(0.17) | 1.80-9.37 | 5 | 0 | <0.01 | 5.00 | 1.00 |
| Robust | Medical history | Kim HJ, 2017 | Isolated single umbilical artery | 4 | 863/99000 | 2.12 | 2.12 | 2.00 | 1.00 | 0.02(0-0.69)(0.38) | 1.31-3.43 | 3 | <0.01 | <0.01 | 3.12 | 0.79 |
| Robust | Medical history | Marshall CA, 2020 | Maternal personality disorder | 5 | 1114/8527780 | 2.98 | 2.65 | 2.50 | 0.16 | 0.26(0-0.72)(0.25) | 1.38-6.44 | 5 | <0.01 | <0.01 | 4.94 | 0.95 |
| Robust | Medical history | Brown NT, 2018 | Sleep-disordered breathing (objective assessment) | 6 | 1695/944724 | 2.32 | 2.32 | 2.28 | 0.68 | 0(0-0.61)(0.53) | 1.87-2.89 | 4 | <0.01 | <0.01 | 4.51 | 0.83 |
| Robust | Obstetric history | Saccone G, 2015 | Prior I-TOP with vacuum aspiration | 5 | 12554/484358 | 1.20 | 1.20 | 1.20 | 0.51 | 0(0-0.64)(0.87) | 1.13-1.27 | 2 | <0.01 | <0.01 | 2.40 | 0.79 |
| Robust | Medical history | Han Z, 2011 | Low gestational weight gain | 3 | 1450795/1243160 | 1.64 | 1.64 | 1.64 | 0.16 | 0(0-0.73)(0.75) | 1.55-1.74 | 1 | 0 | 0 | 1.90 | 0.95 |
| Robust | Medical history | Kangatharan C, 2016 | IPI following miscarriage of <6 months (compared to IPI following miscarriage of ≥6 months, with Conde-Agudelo A, 2004 excluded) | 7 | 27968/32804 | 0.79 | 0.79 | 0.79 | 0.52 | 0(0-0.58)(0.92) | 0.73-0.84 | 2 | <0.01 | <0.01 | 2.77 | 0.83 |
| Highly suggestive | Obstetric history | Saraswat L, 2009 | 1^st^ trimester bleeding | 12 | 74577/13881 | 2.05 | 1.84 | 1.67 | 0.08 | 0.72(0.44-0.83)(<0.01) | 1.27-3.33 | 10 | 0 | <0.01 | 9.18 | 0.44 |
| Highly suggestive | Obstetric history | Saccone G, 2015 | Prior surgical I-TOP (for PTB in singleton gestation) | 10 | 11766/151484 | 1.45 | 1.49 | 1.64 | 0.63 | 0.61(0-0.79)(<0.01) | 0.99-2.12 | 6 | <0.01 | <0.01 | 8.72 | 1.00 |
| Highly suggestive | Obstetric history | Mohan M, 2019 | Obstetric cholestasis | 8 | 9671/2374565 | 3.60 | 3.99 | 2.93 | 0.95 | 0.96(0.94-0.97)(<0.01) | 1.23-10.55 | 6 | 0 | <0.01 | 7.58 | 0.99 |
| Highly suggestive | Obstetric history | Khomami MB, 2018 | PCOS | 32 | 7507/69604 | 1.57 | 1.97 | 2.27 | <0.01 | 0.63(0.43-0.74)(<0.01) | 0.78-3.17 | 10 | 0 | <0.01 | 19.67 | 1.00 |
| Highly suggestive | Medical history | van der Kooi ALF, 2019 | Cancer survivors | 14 | 17495/6070504 | 1.56 | 1.50 | 1.24 | 0.37 | 0.82(0.69-0.88)(<0.01) | 1.01-2.40 | 10 | 0 | <0.01 | 9.18 | 0.44 |
| Highly suggestive | Medical history | Vahanian SA, 2015 | Placenta previa | 9 | 66817/23255950 | 5.32 | 4.59 | 4.52 | 0.20 | 0.98(0.98-0.99)(0) | 2.62-10.81 | 9 | 0 | 0 | 9.00 | 1.00 |
| Highly suggestive | Medical history | Schaaf JM, 2013 | African/black race | 22 | 5876642/25819108 | 1.99 | 2.13 | 1.82 | 0.84 | 1.00(0-1)(0) | 1.36-2.92 | 20 | 0 | 0 | 21.73 | 1.00 |
| Highly suggestive | Medical history | Shah PS, 2011 | Aboriginal ethnicity | 16 | 350784/24335246 | 1.68 | 1.36 | 1.29 | 0.06 | 0.98(0.98-0.98)(0) | 0.96-2.95 | 13 | 0 | <0.01 | 13.70 | 0.80 |
| Highly suggestive | Medical history | Lutsiv O, 2015 | BMI of >40 kg/m^2^ (compared to 30-34.9 kg/m^2^) | 10 | 42603/252510 | 1.30 | 1.23 | 1.19 | 0.44 | 0.70(0.32-0.83)(<0.01) | 1.01-1.70 | 7 | <0.01 | <0.01 | 5.02 | 0.17 |
| Highly suggestive | Medical history | Lutsiv O, 2015 | BMI of >40 kg/m^2^ (compared to 30-39.9 kg/m^2^) | 19 | 61702/454814 | 1.20 | 1.17 | 1.17 | 0.51 | 0.59(0.24-0.74)(<0.01) | 1.00-1.42 | 9 | <0.01 | <0.01 | 9.24 | 0.63 |
| Highly suggestive | Medical history | Lalani S, 2018 | Endometriosis (combined spontaneous conception and assisted reproduction) | 23 | 43262/3019058 | 1.70 | 1.82 | 2.71 | 0.50 | 0.92(0.90-0.94)(0) | 0.74-3.88 | 15 | 0 | <0.01 | 20.49 | 1.00 |
| Highly suggestive | Medical history | Lalani S, 2018 | Endometriosis (spontaneous conception) | 7 | 11264/1435624 | 1.70 | 1.46 | 1.39 | 0.04 | 0.57(0-0.79)(0.03) | 1.01-2.88 | 6 | <0.01 | <0.01 | 3.56 | 0.07 |
| Highly suggestive | Medical history | Leader J, 2018 | Maternal age of ≥45 years old | 9 | 38017/16221724 | 1.96 | 1.56 | 1.58 | 0.21 | 0.91(0.86-0.94)(<0.01) | 1.05-3.65 | 7 | 0 | <0.01 | 7.24 | 0.75 |
| Highly suggestive | Medical history | Zhang JJ, 2015 | CKD during pregnancy | 12 | 898/504746 | 5.63 | 5.30 | 11.08 | 0.87 | 0.87(0.79-0.91)(<0.01) | 0.73-43.51 | 10 | 0 | <0.01 | 11.89 | 1.00 |
| Highly suggestive | Medical history | Han Z, 2011 | Underweight women | 32 | 122813/545378 | 1.21 | 1.25 | 1.25 | 0.32 | 0.80(0.73-0.85)(<0.01) | 0.96-1.53 | 12 | 0 | <0.01 | 17.16 | 0.98 |
| Highly suggestive | Medical history | Zhou SS, 2017 | Maternal vitamin D status (for spontaneous PTB) | 4 | 751/2721 | 1.46 | 1.46 | 1.47 | <0.01 | 0(0-0.58)(1.00) | 1.22-1.74 | 2 | <0.01 | <0.01 | 2.87 | 0.85 |
| Highly suggestive | Medical history | Mengistu TS, 2020 | SMM: haemorrhagic disorders | 8 | 22004/1764888 | 3.43 | 3.12 | 2.40 | 0.71 | 0.96(0.94-0.97)(<0.01) | 1.33-8.85 | 6 | 0 | <0.01 | 8.00 | 1.00 |
| Highly suggestive | Medical history | Mengistu TS, 2020 | SMM: hepatic disorders | 6 | 6733/1216532 | 3.19 | 3.05 | 2.94 | 0.86 | 0.64(0-0.83)(0.02) | 1.55-6.55 | 5 | 0 | <0.01 | 5.44 | 0.90 |
| Highly suggestive | Medical history | Conner SN, 2014 | LEEP | 38 | 6589/369736 | 1.61 | 1.65 | 1.67 | 0.79 | 0.57(0.35-0.70)(<0.01) | 0.98-2.64 | 14 | 0 | <0.01 | 19.66 | 0.98 |
| Highly suggestive | Medical history | Kyrgiou M, 2016 | LLETZ for CIN | 25 | 21296/1423991 | 1.56 | 1.63 | 1.56 | 0.72 | 0.70(0.59-0.77)(<0.01) | 1.02-2.38 | 14 | 0 | <0.01 | 23.11 | 1.00 |
| Highly suggestive | Medical history | Kyrgiou M, 2016 | Any type of treatment for CIN with a cone depth of ≥10-12 mm (compared to untreated CIN) | 8 | 5845/546866 | 1.94 | 1.83 | 1.93 | 0.05 | 0.38(0-0.71)(0.13) | 1.29-2.91 | 7 | <0.01 | <0.01 | 8.00 | 1.00 |
| Highly suggestive | Medical history | Kyrgiou M, 2016 | Any type of treatment for CIN with a cone depth of ≥15-17 mm (compared to untreated CIN) | 4 | 1661/543325 | 2.76 | 2.47 | 2.41 | 0.29 | 0.53(0-0.83)(0.10) | 0.74-10.31 | 4 | <0.01 | <0.01 | 4.00 | 1.00 |
| Highly suggestive | Social and economic profile | Donovan BM, 2016 | Intimate partner violence | 30 | 13013/5060149 | 1.91 | 1.58 | 1.18 | 0.07 | 0.84(0.78-0.87)(<0.01) | 0.86-4.27 | 16 | 0 | <0.01 | 9.49 | 0.01 |
| Highly suggestive | Social and economic profile | Shah PS, 2011 | Unmarried women | 11 | 486952/616314 | 1.22 | 1.23 | 1.27 | 0.68 | 0.90(0.85-0.93)(<0.01) | 0.96-1.55 | 9 | 0 | <0.01 | 9.77 | 0.88 |
| Highly suggestive | Drugs | Gouin K, 2011 | Cocaine | 24 | 3675/36185 | 3.38 | 2.69 | 2.64 | 0.02 | 0.73(0.57-0.81)(<0.01) | 1.43-8.01 | 20 | 0 | <0.01 | 20.38 | 0.71 |
| Highly suggestive | Environmental factors | Liu C, 2017 | Entire pregnancy high-level PM_2.5_ exposure | 3 | NA | 1.06 | 1.04 | 1.08 | 0.15 | 0(0-90)(<0.01) | 0.94-1.20 | 1 | NA | NA | NA | NA |
| Suggestive | Drugs | Tang R, 2019 | Pre-gravid OC use | 4 | 19253/29777 | 1.16 | 1.16 | 1.17 | 0.59 | 0(0-0.68)(0.88) | 0.96-1.41 | 1 | <0.01 | <0.01 | 1.60 | 0.87 |

| Suggestive | Drugs | Conner SN, 2016 | Marijuana during pregnancy | 14 | 4999/76327 | 1.32 | 1.43 | 0.68 | 0.36 | 0.65(0.28-0.79)(<0.01) | 0.84-2.08 | 4 | <0.01 | <0.01 | 10.43 | 1.00 |
| --- | --- | --- | --- | --- | --- | --- | --- | --- | --- | --- | --- | --- | --- | --- | --- | --- |
| Suggestive | Medical history | Mengistu TS, 2020 | SMM: thromboembolic disorders | 3 | 371/1328534 | 2.41 | 2.52 | 2.95 | 0.56 | 0.29(0-0.80)(0.25) | 0.10-60.61 | 2 | <0.01 | <0.01 | 3.00 | 1.00 |
| Suggestive | Infections | Corbella S, 2016 | Periodontal disease | 17 | 1853/6741 | 1.61 | 1.43 | 1.39 | 0.23 | 0.79(0.66-0.86)(<0.01) | 0.80-3.24 | 9 | <0.01 | <0.01 | 6.20 | 0.12 |
| Suggestive | Medical history | Han Z, 2012 | Women of short stature | 9 | 63340/28705 | 1.23 | 1.26 | 1.37 | 0.07 | 0.28(0-0.66)(0.19) | 0.97-1.55 | 3 | <0.01 | <0.01 | 6.93 | 1.00 |
| Suggestive | Drugs | Coughlin CG, 2015 | Antipsychotics during pregnancy | 7 | 2809/1531541 | 1.86 | 1.74 | 1.62 | 0.19 | 0.47(0-0.76)(0.08) | 1.00-3.48 | 3 | <0.01 | <0.01 | 3.92 | 0.86 |
| Suggestive | Infections | Silver BJ, 2014 | *Trichomonas vaginalis* infection | 9 | 2956/78045 | 1.42 | 1.31 | 1.35 | 0.28 | 0.63(0-0.80)(<0.01) | 0.81-2.48 | 4 | <0.01 | <0.01 | 4.58 | 0.76 |
| Suggestive | Assisted reproductive techniques | Alviggi C, 2018 | Blastocyst-stage embryo transfer (vs Cleavage embryo transfer) | 11 | 30618/76011 | 1.14 | 1.23 | 1.29 | <0.01 | 0.54(0-0.74)(<0.01) | 0.94-1.39 | 2 | <0.01 | <0.01 | 7.68 | 1.00 |
| Suggestive | Assisted reproductive techniques | Yang M, 2020 | Fresh blastocyst transfer (for PTB) | 23 | 205919/46225 | 1.24 | 1.20 | 1.10 | 0.41 | 0.72(0.56-0.81)(<0.01) | 0.89-1.73 | 10 | <0.01 | <0.01 | 5.48 | 0.03 |
| Suggestive | Assisted reproductive techniques | Yang M, 2020 | Fresh blastocyst transfer (for very PTB <32 weeks) | 12 | 181873/38716 | 1.27 | 1.27 | 1.24 | 0.18 | 0.10(0-0.54)(0.35) | 1.05-1.53 | 3 | <0.01 | <0.01 | 3.74 | 0.77 |
| Suggestive | Infections | Niyibizi J, 2020 | HPV infection (crude) | 18 | 6498/302076 | 1.70 | 1.33 | 1.02 | <0.01 | 0.71(0.49-0.81)(<0.01) | 0.75-3.85 | 8 | <0.01 | <0.01 | 1.23 | <0.01 |
| Suggestive | Infections | Niyibizi J, 2020 | HPV infection (age-adjusted) | 15 | 5912/266975 | 1.66 | 1.31 | 1.00 | 0.01 | 0.69(0.42-0.81)(<0.01) | 0.69-4.01 | 7 | <0.01 | <0.01 | 0.75 | <0.01 |
| Suggestive | Obstetric history | Saccone G, 2015 | >1 prior surgical I-TOP | 9 | 2209/162876 | 1.98 | 1.96 | 1.66 | 0.90 | 0.82(0.64-0.89)(<0.01) | 0.72-5.46 | 7 | <0.01 | <0.01 | 8.86 | 1.00 |
| Suggestive | Medical history | Kyrgiou M, 2016 | Any type of treatment for CIN with a cone depth of ≥20 mm (compared to untreated CIN) | 3 | 851/542899 | 4.91 | 2.93 | 2.65 | 0.07 | 0.77(0-0.91)(0.01) | <0.01-123653.50 | 3 | <0.01 | <0.01 | 3.00 | 1.00 |
| Weak | Obstetric history | Menzies R, 2020 | History of preterm twins | 6 | 1804/1552 | 4.34 | 4.34 | 3.12 | 0.97 | 0(0-0.61)(0.70) | 2.37-7.95 | 5 | <0.01 | <0.01 | 4.59 | 0.57 |
| Weak | Obstetric history | Menzies R, 2020 | History of preterm twins 34-36+6 weeks | 4 | 823/1170 | 2.13 | 2.13 | 3,46 | 0.54 | 0(0-0.68)(0.60) | 0.62-7.38 | 1 | <0.01 | <0.01 | 2.49 | 0.98 |
| Weak | Obstetric history | Menzies R, 2020 | History of preterm twins 30-33+6 weeks | 3 | 328/1066 | 5.18 | 5.19 | 8.20 | 0.76 | 0.01(0-0.73)(0.36) | 0.08-320.39 | 2 | <0.01 | <0.01 | 2.86 | 0.99 |
| Weak | Obstetric history | Menzies R, 2020 | History of preterm twins <30 weeks | 3 | 313/1066 | 9.73 | 9.72 | 16.22 | 1.00 | 0.28(0-0.80)(0.25) | 0.03-3446.56 | 3 | <0.01 | <0.01 | 3.00 | 1.00 |
| Weak | Obstetric history | Menzies R, 2020 | History of spontaneous twin preterm birth | 4 | 916/1187 | 5.73 | 5.71 | 3.34 | 0.95 | 0.15(0-0.72)(0.32) | 1.19-27.69 | 4 | <0.01 | <0.01 | 3.43 | 0.54 |
| Weak | Obstetric history | Menzies R, 2020 | History of spontaneous twin preterm birth 34-36+6 weeks | 3 | 344/1069 | 2.46 | 2.46 | 1.92 | 0.95 | 0(0-0.73)(0.37) | 0.05-129.70 | 1 | <0.01 | <0.01 | 0.67 | 0.53 |
| Weak | Medical history | Chan YY, 2011 | Subseptate uterus | 3 | 114/2391 | 2.02 | 2.02 | 1.81 | 0.64 | 0(0-0.73)(0.68) | 0.05-74.42 | 1 | 0.01 | 0.01 | 1.43 | 0.86 |
| Weak | Medical history | van den Kooi ALF, 2019 | Cancer survivors treated with radiotherapy | 4 | 1508/32901 | 2.28 | 2.31 | 1.79 | 0.99 | 0.88 (0.67-0.94)(<0.01) | 0.24-21.22 | 3 | <0.01 | <0.01 | 4.00 | 1.00 |
| Weak | Drugs | Etwel F, 2016 | H1 antihistamines | 8 | 1799/9156 | 0.92 | 0.91 | 1.06 | 0.47 | 0.41(0-0.73)(0.10) | 0.43-2.00 | 1 | 0.38 | 0.63 | 0.49 | 0.40 |
| Weak | Medical history | de los Reyes S, 2018 | Velamentous cord insertion | 5 | 15971/1380475 | 1.73 | 1.99 | 2.01 | 0.80 | 0.96(0.94-0.97)(<0.01) | 0.42-7.11 | 5 | 0 | <0.01 | 4.75 | 0.77 |
| Weak | Drugs | Butalia S, 2016 | Metformin | 7 | 754/766 | 1.05 | 1.35 | 1.60 | 0.07 | 0.44(0-0.75)(0.10) | 0.21-5.23 | 2 | 0.10 | 0.89 | 1.20 | 0.35 |
| Weak | Medical history | Xiang LJ, 2018 | Diabetic nephropathy in T1DM | 3 | 155/771 | 4.12 | 4.12 | 4.41 | 0.70 | 0(0-0.73)(0.40) | 0.36-47.37 | 3 | <0.01 | <0.01 | 2.94 | 0.95 |
| Weak | Medical history | Schaaf JM, 2013 | Asian race | 12 | 893496/7418372 | 1.07 | 1.18 | 1.28 | 0.27 | 0.99(0-1)(0) | 0.65-1.76 | 10 | 0 | 0.29 | 12.00 | 1.00 |
| Weak | Medical history | Schaaf JM, 2013 | Hispanic ethnicity | 7 | 5509253/16225595 | 1.02 | 1.20 | 1.21 | 0.25 | 0.99(0.98-0.99)(0) | 0.59-1.75 | 5 | 0 | 0.79 | 6.66 | 1.00 |
| Weak | Medical history | Chakraborty J, 2019 | Laparoscopic appendectomy | 13 | 1770/3848 | 0.61 | 0.59 | 1.45 | 0.78 | 0.45(0-0.70)(0.04) | 0.12-3.17 | 1 | <0.01 | 0.10 | 4.29 | 0.99 |
| Weak | Medical history | Veenendaal MV, 2011 | Hyperemesis gravidarum (cohort studies) | 4 | 3706/1353821 | 1.69 | 1.37 | 1.36 | 0.45 | 0.86(0.56-0.93)(<0.01) | 0.24-11.77 | 2 | <0.01 | 0.02 | 2.46 | 0.84 |
| Weak | Medical history | Veenendaal MV, 2011 | Hyperemesis gravidarum (case-control studies) | 4 | 2567/35476 | 1.14 | 1.25 | 1.40 | 0.10 | 0.43(0-0.80)(0.16) | 0.44-2.95 | 1 | <0.01 | 0.32 | 3.21 | 1.00 |
| Weak | Medical history | Chan YY, 2011 | Arcuate uterus | 7 | 504/5563 | 1.61 | 2.07 | 2.15 | 0.09 | 0.64(0-0.82)(<0.01) | 0.21-12.12 | 2 | <0.01 | 0.17 | 4.24 | 0.98 |

| Weak | Medical history | Chan YY, 2011 | Septate uterus | 4 | 200/729 | 2.30 | 2.30 | 1.88 | 0.65 | 0(0-0.68)(0.52) | 0.85-6.23 | 2 | <0.01 | <0.01 | 1.33 | 0.41 |
| --- | --- | --- | --- | --- | --- | --- | --- | --- | --- | --- | --- | --- | --- | --- | --- | --- |
| Weak | Medical history | Chan YY, 2011 | Bicornuate uterus | 4 | 338/729 | 2.56 | 2.56 | 2.54 | 0.95 | 0(0-0.68)(0.79) | 0.87-7.49 | 2 | <0.01 | <0.01 | 2.71 | 0.91 |
| Weak | Medical history | Chan YY, 2011 | Didelphys uterus | 4 | 131/729 | 3.59 | 3.85 | 5.75 | 0.58 | 0.30(0-0.77)(0.23) | 0.55-23.37 | 2 | <0.01 | <0.01 | 3.14 | 0.97 |
| Weak | Medical history | Chan YY, 2011 | Unicornuate uterus | 5 | 98/2772 | 3.47 | 3.47 | 6.18 | 0.76 | 0(0-0.64)(0.51) | 1.35-8.93 | 1 | <0.01 | <0.01 | 4.37 | 1.00 |
| Weak | Drugs | Marchenko A, 2015 | Triptan | 7 | 3301252450 | 0.97 | 0.81 | 0.85 | 0.22 | 0.68(0.02-0.84)(<0.01) | 0.32-2.93 | 3 | <0.01 | 0.88 | 3.18 | 0.69 |
| Weak | Medical history | Marchenko A, 2015 | Migraine | 3 | 1274/194651 | 1.45 | 1.40 | 1.39 | 0.96 | 0.65(0-0.88)(0.06) | <0.01-9293.04 | 2 | <0.01 | 0.36 | 2.12 | 0.79 |
| Weak | Drugs | Kaplan YC, 2015 | Topical retinoids (exposed infants) | 3 | 377/843 | 0.69 | 1.40 | 0.62 | 0.39 | 0(0-0.73)(0.75) | 0.02-27.66 | 0 | <0.01 | 0.21 | 0.72 | 1.00 |
| Weak | Drugs | Kaplan YC, 2016 | Hydroxychloroquine | 5 | 515/828 | 1.75 | 1.86 | 1.11 | 0.63 | 0.72(0-0.87)(0.01) | 0.21-14.29 | 1 | <0.01 | 0.07 | 0.32 | 0.28 |
| Weak | Infections | Sobhy S, 2016 | TB | 10 | 2729/115295 | 2.68 | 1.52 | 2.32 | 0.40 | 0.67(0.20-0.81)(<0.01) | 0.60-4.69 | 3 | <0.01 | <0.01 | 5.91 | 0.99 |
| Weak | Drugs | Wolf HT, 2017 | Multivitamins | 4 | 26797/15795 | 0.84 | 0.88 | 0.89 | 0.62 | 0.73(0-0.88)(0.01) | 0.35-2.03 | 2 | <0.01 | 0.10 | 1.17 | 0.33 |
| Weak | Medical history | Caissutti C, 2017 | Fetus with small thymus | 3 | 170/437 | 5.15 | 3.50 | 1.07 | 0.35 | 0.81(0-0.92)(<0.01) | <0.01- 11503345664 | 2 | <0.01 | 0.07 | 0.16 | <0.01 |
| Weak | Drugs | Jarde A, 2018 | Probiotics during pregnancy (for PTB <34 weeks) | 6 | 668/576 | 0.88 | 0.88 | 0.33 | <0.01 | 0(0-0.61)(0.86) | 0.18-4.28 | 0 | 0.89 | 0.82 | 0.82 | 1.00 |
| Weak | Drugs | Jarde A, 2018 | Probiotics during pregnancy (for PTB <37 weeks) | 11 | 1215/1158 | 1.10 | 1.10 | 1.29 | 0.78 | 0(0-0.53)(0.64) | 0.67-1.81 | 0 | 0.65 | 0.65 | 0.69 | 1.00 |
| Weak | Social and economic profile | Liu N, 2019 | Home visits for pregnant women | 7 | 2770/3007 | 0.93 | 0.96 | 1.07 | 0.26 | 0.28(0-0.70)(0.22) | 0.59-1.45 | 1 | 0.62 | 0.46 | 0.46 | 0.38 |
| Weak | Medical history | Liu L, 2019 | APS | 5 | 688/212039 | 1.90 | 1.92 | 1.93 | 0.88 | 0.49(0-0.80)(0.09) | 0.53-6.76 | 2 | <0.01 | <0.01 | 2.94 | 0.90 |
| Weak | Medical history | Matenchuk B, 2019 | Bed Rest (in developing regions, for PTB <37 weeks) | 5 | 353/353 | 0.92 | 0.92 | 0.91 | 0.97 | 0.55(0-0.81)(0.06) | 0.46-1.82 | 1 | 0.19 | 0.45 | 0.29 | 0.26 |
| Weak | Medical history | Matenchuk B, 2019 | Bed Rest (in developed regions, for PTB <37 weeks) | 10 | 692/1113 | 1.02 | 1.02 | 1.00 | 0.27 | 0(0-0.53)(0.95) | 0.91-1.13 | 0 | 0.72 | 0.72 | 0.50 | 1.00 |
| Weak | Medical history | Matenchuk B, 2019 | Bed Rest (in developing regions, for very PTB) | 5 | 353/353 | 0.90 | 0.90 | 0.90 | 0.88 | 0(0-0.64)(0.86) | 0.43-1.88 | 0 | 0.64 | 0.64 | 0.27 | 1.00 |
| Weak | Medical history | Matenchuk B, 2019 | Bed Rest (in developed regions, for very PTB) | 4 | 144/149 | 2.07 | 2.07 | 1.75 | 0.55 | 0(0-0.68)(0.89) | 0.57-7.53 | 0 | 0.02 | 0.02 | 0.50 | 1.00 |
| Weak | Infections | Thompson JM, 2019 | Pregnancy-associated malaria | 8 | 862/3305 | 1.22 | 1.22 | 1.12 | 0.43 | 0(0-0.56)(0.64) | 1.03-1.46 | 1 | <0.01 | <0.01 | 0.67 | 0.50 |
| Weak | Drugs | Taylor L, 2020 | Nicotine replacement therapy | 5 | 976/979 | 0.74 | 0.78 | 0.91 | 0.10 | 0.30(0-0.74)(0.22) | 0.29-1.89 | 1 | 0.09 | 0.10 | 0.30 | 0.27 |
| Weak | Medical history | Amezcua-Prieto C, 2020 | Women involved in motor vehicle crashes | 3 | 27003/1774801 | 2.02 | 1.07 | 0.96 | 0.37 | 0.99(0.99-1.00)(0) | <0.01-1134171.88 | 2 | <0.01 | 0.18 | 2.12 | 0.79 |
| Weak | Assisted reproductive techniques | Allen CP, 2020 | Donor sperm (for PTB) | 12 | 23291/5229762 | 0.97 | 0.95 | 0.97 | 0.97 | 0.54(0-0.74)(0.01) | 0.75-1.26 | 2 | 0.03 | 0.61 | 3.25 | 0.88 |
| Weak | Assisted reproductive techniques | Allen CP, 2020 | Donor sperm (for very PTB) | 5 | 9020/665950 | 0.93 | 0.93 | 0.85 | 0.99 | 0(0-0.64)(0.53) | 0.71-1.22 | 0 | 0.39 | 0.39 | 4.13 | 1.00 |
| Weak | Medical history | Yi XY, 2015 | Bariatric surgery | 7 | 621/2551 | 1.18 | 1.18 | 1.09 | 0.97 | 0(0-0.58)(0.68) | 0.79-1.78 | 0 | 0.29 | 0.29 | 0.41 | 1.00 |
| Weak | Supplements | Rumbold A, 2016 | Vitamin C and others supplementation | 16 | 11095/11155 | 0.99 | 1.00 | 0.97 | 0.54 | 0.49(0-0.70)(0.02) | 0.75-1.32 | 4 | 0.90 | 0.92 | 0.89 | 0.01 |
| Weak | Medical history | Brown NT, 2018 | Sleep-disordered breathing (questionnaire-based assessment) | 11 | 2086/7355 | 1.58 | 1.50 | 1.23 | 0.63 | 0.48(0-0.72)(0.04) | 0.74-3.39 | 3 | <0.01 | <0.01 | 2.44 | 0.45 |
| Weak | Medical history | Murphy VE, 2015 | Asthma with exacerbation during pregnancy | 4 | 126/31522 | 1.45 | 1.45 | 1.76 | 0.49 | 0(0-0.68)(0.84) | 0.36-5.89 | 0 | 0.26 | 0.26 | 1.82 | 1.00 |
| Weak | Medical history | Murphy VE, 2015 | Asthma without exacerbation during pregnancy | 4 | 1312/31899 | 0.97 | 0.93 | 0.78 | 0.11 | 0.22(0-0.74)(0.28) | 0.42-2.24 | 0 | 0.52 | 0.81 | 1.43 | 1.00 |
| Weak | Drugs | Patra J, 2011 | Alcohol consumption before or during pregnancy | 4 | 9742/79074 | 1.37 | 1.67 | 1.86 | 0.25 | 0.85(0.51-0.92)(<0.01) | 0.27-6.90 | 2 | <0.01 | 0.09 | 3.99 | 1.00 |
| Weak | Drugs | Reboucas KF, 2019 | Vaginal clindamycin treatment for bacterial vaginosis | 6 | 1096/1105 | 1.00 | 1.01 | 1.13 | 0.88 | 0.15(0-0.67)(0.32) | 0.50-2.00 | 0 | 0.95 | 0.98 | 0.39 | 1.00 |

| Weak | Assisted reproductive techniques | Kamath MS, 2018 | Stimulated cycle IVF | 4 | 96996/702 | 1.27 | 1.27 | 1.35 | 0.14 | 0(0-0.68)(0.58) | 0.79-2.04 | 1 | 0.03 | 0.03 | 1.55 | 0.86 |
| --- | --- | --- | --- | --- | --- | --- | --- | --- | --- | --- | --- | --- | --- | --- | --- | --- |
| Weak | Infections | Leitich H, 2007 | Bacterial vaginosis | 16 | 1391/8993 | 1.80 | 1.86 | 7.55 | 0.79 | 0.72(0.50-0.82)(<0.01) | 0.43-7.43 | 4 | <0.01 | <0.01 | 15.90 | 1.00 |
| Weak | Infections | Leitich H, 2007 | Intermediate vaginal flora | 5 | 213/1635 | 2.46 | 3.49 | 6.01 | 0.09 | 0.66(0-0.85)(0.02) | 0.11-56.89 | 2 | <0.01 | 0.07 | 4.92 | 1.00 |
| Weak | Vaccines | Wang A, 2019 | HPV 6/11/16/18 vaccine in periconceptional period or during pregnancy | 3 | 4579/16716 | 1.08 | 1.08 | 1.01 | 0.83 | 0(0-0.73)(0.68) | 0.44-2.62 | 0 | 0.30 | 0.30 | 0.15 | 1.00 |
| Weak | Drugs | Ziv A, 2018 | Quinolones during 1^st^ trimester | 4 | 1678/269927 | 1.10 | 1.05 | 0.86 | 0.37 | 0.42(0-0.80)(0.16) | 0.39-3.11 | 0 | 0.62 | 0.51 | 3.07 | 1.00 |
| Weak | Drugs | Morency AM, 2007 | Macrolides | 3 | 1014/793 | 0.70 | 0.72 | 0.62 | 0.67 | 0.55(0-0.86)(0.11) | 0.01-47.57 | 2 | 0.01 | 0.08 | 1.92 | 0.70 |
| Weak | Drugs | Morency AM, 2007 | Clindamycin | 5 | 764/759 | 0.75 | 0.69 | 0.47 | 0.45 | 0.63(0-0.84)(0.03) | 0.11-5.18 | 2 | 0.03 | 0.33 | 2.79 | 0.88 |
| Weak | Drugs | Morency AM, 2007 | Metronidazole alone or in combination | 8 | 2779/2531 | 1.29 | 1.09 | 0.97 | 0.05 | 0.76(0.42-0.86)(<0.01) | 0.43-3.88 | 5 | 0.28 | 0.15 | 0.42 | <0.01 |
| Weak | Drugs | Morency AM, 2007 | Metronidazole | 6 | 2006/1985 | 1.58 | 1.29 | 0.97 | 0.04 | 0.70(0-0.85)(0.01) | 0.46-5.47 | 4 | <0.01 | 0.02 | 0.31 | <0.01 |
| Weak | Infections | Wagle M, 2018 | Dental caries | 5 | 2187/2059 | 1.16 | 1.16 | 1.17 | 0.83 | 0.35(0-0.75)(0.19) | 0.60-2.27 | 1 | 0.04 | 0.24 | 0.97 | 0.66 |
| Weak | Medical history | Tersigni C, 2014 | Celiac disease | 3 | 2854/3295900 | 1.41 | 1.39 | 1.46 | 0.43 | 0.48(0-0.84)(0.15) | 0.16-12.84 | 3 | <0.01 | <0.01 | 2.18 | 0.38 |
| Weak | Medical history | Ong SS, 2006 | Single-twin death after 14 weeks of monochorionic pregnancy | 6 | 45/23 | 1.86 | 1.88 | 6.50 | 0.44 | 0.04(0-0.62)(0.39) | 0.31-11.27 | 0 | 0.27 | 0.29 | 1.89 | 1.00 |
| Weak | Medical history | Carter EB, 2016 | Prenatal care (observational studies) | 7 | 1251/4811 | 0.93 | 0.85 | 0.62 | 0.21 | 0.48(0-0.76)(0.08) | 0.35-2.52 | 1 | 0.21 | 0.72 | 2.46 | 0.95 |
| Weak | Medical history | Carter EB, 2016 | Prenatal care (randomized clinical trials) | 4 | 1539/1262 | 0.83 | 0.82 | 0.71 | 0.66 | 0.23(0-0.75)(0.27) | 0.36-1.90 | 0 | 0.09 | 0.17 | 1.25 | 1.00 |
| Weak | Medical history | Lalani S, 2018 | Endometriosis (assisted reproduction) | 10 | 3072/20600 | 1.27 | 1.23 | 1.24 | 0.52 | 0.41(0-0.70)(0.08) | 0.77-2.08 | 4 | <0.01 | 0.02 | 2.63 | 0.25 |
| Weak | Medical history | Berghella V, 2013 | Knowledge of TVU-measured CL in singleton pregnancies with symptoms of PTL | 3 | 129/127 | 0.55 | 0.55 | 0.57 | 0.73 | 0(0-0.73)(0.97) | <0.01-84.58 | 0 | 0.13 | 0.13 | 0.40 | 1.00 |
| Weak | Obstetric history | Saccone G, 2015 | Only 1 prior surgical I-TOP | 23 | 142543/732698 | 1.53 | 1.57 | 1.20 | 0.74 | 0.99(0-1.00)(0) | 0.19-12.52 | 12 | 0 | 0.04 | 11.21 | 0.45 |
| Weak | Obstetric history | Saccone G, 2015 | Prior 1^st^ trimester surgical I-TOP | 5 | 6083/88013 | 2.36 | 7.43 | 19.51 | 0.41 | 1.00(1.00-1.00)(0) | <0.01-1723.75 | 2 | 0 | 0.32 | 5.00 | 1.00 |
| Weak | Obstetric history | Saccone G, 2015 | Prior S-TOP | 5 | 10748/113385 | 1.19 | 1.19 | 1.16 | 0.56 | 0.60(0-0.83)(0.04) | 0.77-1.83 | 3 | <0.01 | 0.02 | 2.66 | 0.56 |
| Weak | Obstetric history | Saccone G, 2015 | Prior uterine evacuation | 34 | 161123/871177 | 1.44 | 1.51 | 1.20 | 0.81 | 0.99(0-1.00)(0) | 0.28-7.40 | 17 | 0 | <0.01 | 16.31 | 0.47 |
| Weak | Obstetric history | Saccone G, 2015 | Prior I-TOP | 29 | 149395/757792 | 1.52 | 1.57 | 1.20 | 0.74 | 0.99(0-1.00)(0) | 0.24-9.83 | 14 | 0 | 0.01 | 13.32 | 0.47 |
| Weak | Obstetric history | Saccone G, 2015 | Prior surgical I-TOP (for PTB in general) | 27 | 149221/757076 | 1.52 | 1.57 | 1.20 | 0.73 | 0.99(0-1.00)(0) | 0.23-10.01 | 14 | 0 | 0.02 | 13.01 | 0.42 |
| Weak | Obstetric history | Saccone G, 2015 | Prior I-TOP with dilation and evacuation | 5 | 6232/62447 | 1.39 | 1.29 | 1.04 | 0.35 | 0.64(0-0.84)(0.03) | 0.63-3.06 | 2 | <0.01 | 0.01 | 0.41 | 0.06 |
| Weak | Medical history | Sheehan PM, 2015 | Hyperthyroidism | 5 | 21879/1617856 | 1.25 | 1.24 | 1.24 | 0.84 | 0.28(0-0.73)(0.23) | 0.42-3.75 | 1 | <0.01 | 0.30 | 2.19 | 0.94 |
| Weak | Medical history | Nasirkandy PM, 2017 | Clinical hypothyroidism | 10 | 20076/2452817 | 1.31 | 1.20 | 1.27 | 0.57 | 0.63(0.07-0.80)(<0.01) | 0.82-2.09 | 4 | <0.01 | 0.01 | 3.81 | 0.57 |
| Weak | Medical history | Nasirkandy PM, 2017 | Subclinical hypothyroidism | 17 | 3580/64785 | 1.36 | 1.29 | 1.27 | 0.38 | 0.45(0-0.68)(0.02) | 0.73-2.53 | 5 | <0.01 | <0.01 | 6.49 | 0.84 |
| Weak | Medical history | Nasirkandy PM, 2017 | Hypothyroxinemia | 7 | 1078/44377 | 1.30 | 1.32 | 1.27 | 0.22 | 0.20(0-0.66)(0.28) | 0.74-2.27 | 1 | 0.02 | 0.06 | 3.78 | 1.00 |
| Weak | Drugs | Sun X, 2020 | LT4 treatment in euthyroid women with thyroid autoimmunity (with Negro R, 2016 included) | 5 | 669/681 | 0.79 | 0.83 | 1.20 | 0.31 | 0.29(0-0.74)(0.22) | 0.25-2.46 | 1 | 0.30 | 0.30 | 0.37 | 0.32 |
| Weak | Drugs | Sun X, 2020 | LT4 treatment in euthyroid women with thyroid autoimmunity (with Negro R, 2016 excluded) | 4 | 471/486 | 0.81 | 0.90 | 1.20 | 0.33 | 0.41(0-0.80)(0.16) | 0.10-6.67 | 1 | 0.62 | 0.49 | 0.28 | 0.25 |
| Weak | Medical history | Shah PS, 2010 | Primiparous mother | 6 | 487369/509293 | 1.13 | 1.14 | 1.00 | 0.96 | 0.99(0.98-0.99)(0) | 0.62-2.07 | 3 | 0 | 0.15 | 0.30 | <0.01 |
| Weak | Medical history | McDonald SD, 2011 | High gestational weight gain | 3 | 89418/1243160 | 0.76 | 0.72 | 0.73 | 0.68 | 0.16(0-0.77)(0.30) | 0.07-7.65 | 1 | 0 | 0.02 | 1.28 | 0.81 |
| Weak | Obstetric history | Kangatharan C, 2016 | IPI following miscarriage of <6 months (compared to IPI following miscarriage of ≥6 months, with Conde-Agudelo A, 2004 included) | 8 | 39583/279297 | 0.93 | 1.48 | 2.03 | 0.28 | 0.99(0.99-0.99)(0) | 0.18-4.76 | 3 | 0 | 0.74 | 6.78 | 1.00 |
| Weak | Obstetric history | Kangatharan C, 2016 | IPI following miscarriage of <6 months (compared to IPI following miscarriage of 6-12 months) | 5 | 38762/52812 | 1.10 | 1.72 | 2.23 | 0.30 | 0.99(0.99-0.99)(0) | 0.14-8.37 | 2 | 0 | 0.73 | 4.61 | 1.00 |
| Weak | Obstetric history | Kangatharan C, 2016 | IPI following miscarriage of <6 months (compared to IPI following miscarriage of >12 months) | 6 | 40706/227281 | 1.05 | 1.80 | 2.43 | 0.21 | 0.99(0.99-1.00)(0) | 0.12-9.54 | 3 | 0 | 0.87 | 5.72 | 1.00 |

| Weak | Medical history | Danhof NA, 2015 | Treated CIN (for PTB <37 weeks) | 15 | 20884/64237 | 1.66 | 3.32 | 3.99 | 0.02 | 0.95(0.94-0.96)(0) | 0.26-10.57 | 6 | 0 | 0.03 | 12.83 | 1.00 |
| --- | --- | --- | --- | --- | --- | --- | --- | --- | --- | --- | --- | --- | --- | --- | --- | --- |
| Weak | Medical history | Danhof NA, 2015 | Treated CIN during pregnancy | 3 | 52/458 | 6.43 | 8.06 | 7.50 | 0.33 | 0.55(0-0.86)(0.11) | <0.01-485648192 | 2 | <0.01 | 0.03 | 2.74 | 0.98 |
| Weak | Medical history | Danhof NA, 2015 | Treated CIN before pregnancy | 13 | 20832/64162 | 1.39 | 3.31 | 3.99 | 0.01 | 0.96(0.95-0.97)(0) | 0.21-9.20 | 4 | 0 | 0.19 | 11.69 | 1.00 |
| Weak | Medical history | Danhof NA, 2015 | Untreated CIN | 5 | 13754/8902865 | 1.51 | 1.08 | 0.97 | 0.08 | 0.91(0.83-0.95)(<0.01) | 0.42-5.49 | 2 | 0.02 | 0.02 | 1.51 | 0.48 |
| Weak | Medical history | Danhof NA, 2015 | Treated CIN (for spontaneous PTB <37 weeks) | 5 | 4331/5914 | 0.88 | 1.09 | 1.32 | 0.18 | 0.67(0-0.85)(0.02) | 0.18-4.29 | 2 | 0.44 | 0.60 | 1.60 | 0.51 |
| Weak | Medical history | Danhof NA, 2015 | Treated CIN (for PTB <32 weeks) | 5 | 17206/58981 | 1.69 | 3.25 | 3.44 | 0.08 | 0.85(0.60-0.92)(<0.01) | 0.14-21.00 | 1 | 0 | 0.16 | 4.23 | 1.00 |
| Weak | Medical history | Zhou SS, 2017 | Maternal 25-OHD concentration of <50 nmol/L | 16 | 2566/382139 | 1.21 | 1.25 | 1.44 | 0.76 | 0.39(0-0.65)(0.05) | 0.78-1.87 | 4 | <0.01 | 0.03 | 8.51 | 0.99 |
| Weak | Medical history | Zhou SS, 2017 | Maternal 25-OHD concentration of <75 nmol/L | 15 | 2529/14593 | 1.09 | 1.14 | 1.34 | 0.62 | 0.66(0.34-0.79)(0) | 0.55-2.18 | 3 | <0.01 | 0.41 | 7.15 | 0.99 |
| Weak | Supplements | Zhou SS, 2017 | Vitamin D supplementation | 6 | 914/773 | 0.52 | 0.58 | 0.61 | 0.43 | 0.41(0-0.75)(0.13) | 0.08-3.23 | 2 | 0.03 | 0.08 | 0.93 | 0.23 |
| Weak | Medical history | Zhou SS, 2017 | Maternal vitamin D status  (for PTB in general) | 13 | 2436/14036 | 1.20 | 1.24 | 1.44 | 0.74 | 0.51(0-0.73)(0.02) | 0.71-2.02 | 4 | <0.01 | 0.06 | 8.04 | 0.99 |
| Weak | Medical history | Kyrgiou M, 2016 | Any type of treatment for CIN with a cone depth of ≤10-12 mm (compared to untreated CIN) | 8 | 4105/546824 | 1.55 | 1.28 | 1.04 | 0.26 | 0.67(0.10-0.83)(<0.01) | 0.60-3.99 | 3 | <0.01 | 0.01 | 8.00 | 1.00 |
| Weak | Medical history | Kyrgiou M, 2016 | Any type of treatment for CIN with a cone depth of ≥10-12 mm (compared to any type of treatment for CIN with a cone depth of ≤10-12 mm) | 7 | 3276/3083 | 1.54 | <0.01 | 1.55 | 0.91 | 0(0-0.58)(0.48) | 1.25-1.89 | 2 | <0.01 | <0.01 | 7.00 | 1.00 |
| Weak | Medical history | Kyrgiou M, 2016 | Any type of treatment for CIN with a cone depth of ≥15-17 mm (compared to any type of treatment for CIN with a cone depth of ≤15-17 mm) | 4 | 1661/2614 | 1.83 | 1.83 | 1.74 | 0.08 | 0(0-0.68)(0.55) | 1.14-2.94 | 3 | <0.01 | <0.01 | 4.00 | 1.00 |
| Weak | Medical history | Kyrgiou M, 2016 | Any type of treatment for CIN with a cone depth of ≥20 mm (compared to any type of treatment for CIN with a cone depth of ≤20 mm) | 3 | 851/3093 | 2.79 | 1.90 | 1.67 | 0.16 | 0.63(0-0.87)(0.07) | <0.01-19703.54 | 3 | <0.01 | 0.01 | 3.00 | 1.00 |
| Weak | Environmental factors | Liu C, 2017 | 1^st^ trimester PM_2.5_ exposure | 9 | NA | 1.15 | 1.11 | 1.10 | 0.45 | 0.34(0-0.69)(0.15) | 0.76-1.72 | 4 | NA | NA | NA | NA |
| Weak | Environmental factors | Liu C, 2017 | Entire pregnancy PM_2.5_ exposure | 7 | NA | 1.15 | 1.07 | 1.06 | 0.45 | 0.84(0.67-0.91)(<0.01) | 0.62-2.01 | 4 | NA | NA | NA | NA |
| Weak | Environmental factors | Liu C, 2017 | 1^st^ trimester high-level PM_2.5_ exposure | 4 | NA | 1.12 | 1.12 | 1.00 | 0.75 | 0.38(0-0.79)(0.18) | 0.62-2.01 | 1 | NA | NA | NA | NA |
| Weak | Environmental factors | Liu C, 2017 | 1^st^ trimester low-level PM_2.5_ exposure | 5 | NA | 1.17 | 1.11 | 1.10 | 0.37 | 0.45(0-0.78)(0.12) | 0.84-1.62 | 3 | NA | NA | NA | NA |
| Weak | Environmental factors | Liu C, 2017 | Entire pregnancy low-level PM_2.5_ exposure | 4 | NA | 1.31 | 1.06 | 1.63 | 0.45 | 0.47(0-82)(0.13) | 0.58-2.96 | 3 | NA | NA | NA | NA |
| Weak | Environmental factors | Sun X, 2015 | Entire pregnancy PM_2.5_ exposure | 13 | NA | 1.11 | 1.04 | 0.97 | 0.04 | 0.86(0.77-0.90)(<0.01) | 0.87-1.42 | 5 | NA | NA | NA | NA |
| Weak | Environmental factors | Sun X, 2015 | 1^st^ trimester PM_2.5_ exposure | 10 | NA | 1.08 | 1.01 | 1.02 | 0.49 | 0.90(0.84-0.93)(<0.01) | 0.66-1.77 | 4 | NA | NA | NA | NA |
| Weak | Environmental factors | Sun X, 2015 | 2^nd^ trimester PM_2.5_ exposure | 5 | NA | 1.10 | 1.10 | 1.06 | 0.79 | 0.97(0.96-0.98)(<0.01) | 0.54-2.27 | 3 | NA | NA | NA | NA |
| Weak | Environmental factors | Sun X, 2015 | 3^rd^ trimester PM_2.5_ exposure | 9 | NA | 1.06 | 1.01 | 1.00 | 0.16 | 0.81(0.61-0.88)(<0.01) | 0.90-1.25 | 4 | NA | NA | NA | NA |
| Weak | Environmental factors | Sun X, 2015 | 1^st^ month PM_2.5_ exposure | 3 | NA | 1.10 | 1.03 | 1.00 | 0.65 | 0.92(0.76-0.96)(<0.01) | 0.16-7.58 | 1 | NA | NA | NA | NA |
| Weak | Environmental factors | Sun X, 2015 | Within 1 month before birth PM_2.5_ exposure | 6 | NA | 0.99 | 1.00 | 1.00 | 0.99 | 0.90(0.82-0.94)(<0.01) | 0.73-1.34 | 2 | NA | NA | NA | NA |
| Weak | Environmental factors | Sun X, 2015 | Individual-level PM_2.5_ exposure | 3 | NA | 1.10 | 1.02 | 1.00 | 0.33 | 0.58(0-0.86)(0.09) | 0.13-9.23 | 1 | NA | NA | NA | NA |
| Weak | Environmental factors | Sun X, 2015 | Semi-individual-level PM_2.5_ exposure | 9 | NA | 1.15 | 1.12 | 1.06 | 0.78 | 0.86(0.74-0.91)(<0.01) | 0.78-1.68 | 5 | NA | NA | NA | NA |
| Weak | Environmental factors | Sun X, 2015 | Regional-level PM_2.5_ exposure | 4 | NA | 1.07 | 1.07 | 1.04 | 0.93 | 0.93(0.86-0.96)(<0.01) | 0.58-1.99 | 1 | NA | NA | NA | NA |
| Weak | Environmental factors | Sun X, 2015 | PM_2.5_ exposure | 26 | NA | 1.11 | 1.04 | 0.97 | <0.01 | 0.85(0.80-0.89)(<0.01) | 0.90-1.37 | 10 | NA | NA | NA | NA |
| Weak | Environmental factors | Simoncic V, 2020 | 1^st^, 2^nd^ or 3^rd^ trimester NO_2_ exposure | 8 | NA | 1.06 | 0.98 | 0.96 | 0.04 | 0.68(0.11-0.83)(<0.01) | 0.84-1.33 | 1 | NA | NA | NA | NA |
| Weak | Environmental factors | Simoncic V, 2020 | Whole pregnancy NO_2_ exposure | 4 | NA | 1.07 | 0.99 | 0.96 | 0.44 | 0.72(0-0.88)(0.01) | 0.53-2.19 | 1 | NA | NA | NA | NA |

**Additional file 2.** Details of the comparisons

Abbreviations: Random effects, summary odds ratio or risk ratio using random effects model; Fixed effects, summary odds ratio or risk ratio using fixed effects model; Largest effect, odds ratio or risk ratio of the largest study in the meta-analysis; Egger, p-value from Egger's regression asymmetry test for evaluation of publication bias; O, observed number of "positive" studies; P, p-value; E, expected number of "positive" studies; NA, non-applicable; I-TOP, induced termination of pregnancy; PTB, preterm birth; S-TOP, spontaneous termination of pregnancy; PCOS, polycystic ovary syndrome; APS, antiphospholipid syndrome; T1DM, type 1 diabetes mellitus; SMM, severe maternal morbidity; BMI, body mass index; TVU, transvaginal ultrasound; CL, cervical length; PTL, preterm labor; CKD, chronic kidney disease; CIN, cervical intraepithelial neoplasia; 25-OHD, 25-hydroxyvitamin D; LEEP, loop electrosurgical excision procedure; LLETZ, large loop excision of transformation zone; OC, oral contraceptive; LT4, levothyroxine; HPV, human papillomavirus; TB, tuberculosis; IVF, in vitro fertilization; PM2.5, particulate matter with aerodynamic diameter less than or equal to 2.5 μm

* Summary random effects odds ratio or risk ratio of each meta-analysis

† Summary fixed effects odds ratio or risk ratio of each meta-analysis

‡ Odds ratio or risk ratio of the largest study in each meta-analysis

§ P-value from the Egger regression asymmetry test for evaluation of publication bias

|| I2 metric of inconsistency (95% confidence intervals of I2) and P-value of the Cochran Q test for evaluation of heterogeneity

≠ 95% Prediction Interval

¶ Observed number of statistically significant studies

** P-value of the excess statistical significance test

ȣ Expected number of statistically significant studies using the effect of the largest study of each meta-analysis as the plausible effect size
