# Supplementary material for: Risk factors for preterm birth: an umbrella review of meta-analyses of observational studies
Source: BMC Med. 2023 Dec 13;21:494. doi: 10.1186/s12916-023-03171-4 (PMC10720103; doi:10.1186/s12916-023-03171-4)
Supplement: Supplementary file 3 — Additional file 3. Details of the comparisons with robust evidence. [file 12916_2023_3171_MOESM3_ESM.docx]

| Level of Evidence | Area | Author, year | Comparison | Studies | Cases/controls | Random effects* | Fixed effects**†** | Largest effect**‡** | Egger§ | I2(95%CI)(P)**\|\|** | 95% PI**≠** | O**¶** | P**  (fixed) | P**  (random) | E  (largest)ȣ | P**  (largest) |
| --- | --- | --- | --- | --- | --- | --- | --- | --- | --- | --- | --- | --- | --- | --- | --- | --- |
| Robust | Obstetric history | Saccone G, 2015 | Prior I-TOP with vacuum aspiration | 5 | 12554/484358 | 1.20 | 1.20 | 1.20 | 0.51 | 0(0-0.64)(0.87) | 1.13-1.27 | 2 | <0.01 | <0.01 | 2.40 | 0.79 |
| Robust | Medical history | Kim HJ, 2017 | Fetus with isolated single umbilical artery | 4 | 863/99000 | 2.12 | 2.12 | 2.00 | 1.00 | 0.02(0-0.69)(0.38) | 1.31-3.43 | 3 | <0.01 | <0.01 | 3.12 | 0.79 |
| Robust | Medical history | Marshall CA, 2020 | Maternal personal disorder | 5 | 1114/8527780 | 2.98 | 2.65 | 2.50 | 0.16 | 0.26(0-0.72)(0.25) | 1.38-6.44 | 5 | <0.01 | <0.01 | 4.94 | 0.95 |
| Robust | Medical history | Brown NT, 2018 | Sleep-disordered breathing (objective assessment) | 6 | 1695/944724 | 2.32 | 2.32 | 2.28 | 0.68 | 0(0-0.61)(0.53) | 1.87-2.89 | 4 | <0.01 | <0.01 | 4.51 | 0.83 |
| Robust | Medical history | Kangatharan C, 2016 | IPI following miscarriage of <6 months (compared to IPI following miscarriage of ≥6 months, with Conde-Agudelo A, 2004 excluded) | 7 | 27968/32804 | 0.79 | 0.79 | 0.79 | 0.52 | 0(0-0.58)(0.92) | 0.73-0.84 | 2 | <0.01 | <0.01 | 2.77 | 0.83 |
| Robust | Medical history | Han Z, 2011 | Low gestational weight gain | 3 | 1450795/1243160 | 1.64 | 1.64 | 1.64 | 0.16 | 0(0-0.73)(0.75) | 1.55-1.74 | 1 | 0 | 0 | 1.90 | 0.95 |
| Robust | Drugs | Ladhani NN, 2011 | Amphetamines | 5 | 641/61429 | 4.11 | 4.56 | 5.24 | 0.12 | 0.38(0-0.76)(0.17) | 1.80-9.37 | 5 | 0 | <0.01 | 5.00 | 1.00 |

**Additional file 3.** Details of the comparisons with robust evidence

**Additional file 3.** Details of the comparisons with robust evidence

Abbreviations: Random effects, summary odds ratio or risk ratio using random effects model; Fixed effects, summary odds ratio or risk ratio using fixed effects model; Largest effect, odds ratio or risk ratio of the largest study in the meta-analysis; Egger, p-value from Egger's regression asymmetry test for evaluation of publication bias; O, observed number of "positive" studies; P, p-value; E, expected number of "positive" studies; I-TOP: induced termination of pregnancy.

* Summary random effects odds ratio or risk ratio of each meta-analysis

† Summary fixed effects odds ratio or risk ratio of each meta-analysis

‡ Odds ratio or risk ratio of the largest study in each meta-analysis

§ P-value from the Egger regression asymmetry test for evaluation of publication bias

|| I2 metric of inconsistency (95% confidence intervals of I2) and P-value of the Cochran Q test for evaluation of heterogeneity

≠ 95% Prediction Interval

¶ Observed number of statistically significant studies

** P-value of the excess statistical significance test

ȣ Expected number of statistically significant studies using the effect of the largest study of each meta-analysis as the plausible effect size
