# Supplementary material for: Risk factors for preterm birth: an umbrella review of meta-analyses of observational studies
Source: BMC Med. 2023 Dec 13;21:494. doi: 10.1186/s12916-023-03171-4 (PMC10720103; doi:10.1186/s12916-023-03171-4)
Supplement: Supplementary file 4 — Additional file 4. Details of the protective factors. [file 12916_2023_3171_MOESM4_ESM.docx]

| **Additional file 4.** Details of the protective factors  **Abbreviations:** Random effects, summary odds ratio or risk ratio using random effects model; Fixed effects, summary odds ratio or risk ratio using fixed effects model; Largest effect, odds ratio or risk ratio of the largest study in the meta-analysis; Egger, p-value from Egger's regression asymmetry test for evaluation of publication bias; O, observed number of "positive" studies; P, p-value; E, expected number of "positive" studies; NA, non-applicable; IPI, interpregnancy interval | | | | | | | |
| --- | --- | --- | --- | --- | --- | --- | --- |
| * Summary random effects odds ratio or risk ratio of each meta-analysis | | | | | |  |  |
| † Summary fixed effects odds ratio or risk ratio of each meta-analysis | | | | | |  |  |
| ‡ Odds ratio or risk ratio of the largest study in each meta-analysis | | | | | |  |  |
| § P-value from the Egger regression asymmetry test for evaluation of publication bias | | | | | | |  |
| \|\| I^2^ metric of inconsistency (95% confidence intervals of I^2^) and P-value of the Cochran Q test for evaluation of heterogeneity  **≠** 95% Prediction Interval | | | |  |  |  |  |
| ¶ Observed number of statistically significant studies |  |  |  |  |  |  |  |
| ** P-value of the excess statistical significance test |  |  |  |  |  |  |  |
| ȣ Expected number of statistically significant studies using the effect of the largest study of each meta-analysis as the plausible effect size | | | | |  |  |  |

| Level of Evidence | Area | Author, year | Comparison | Studies | Cases/controls | Random effects* | Fixed effects**†** | Largest effect**‡** | Egger§ | I2(95%CI)(P)**\|\|** | 95% PI**≠** | O**¶** | P**  (fixed) | P**  (random) | E  (largest)ȣ | P**  (largest) |
| --- | --- | --- | --- | --- | --- | --- | --- | --- | --- | --- | --- | --- | --- | --- | --- | --- |
| Robust | Obstetric history | Kangatharan C, 2016 | IPI following miscarriage of <6 months (compared to IPI following miscarriage of ≥6 months, with Conde-Agudelo A, 2004 excluded) | 7 | 27968/32804 | 0.79 | 0.79 | 0.79 | 0.52 | 0(0-0.58)(0.92) | 0.73-0.84 | 2 | <0.01 | <0.01 | 2.77 | 0.83 |
| Suggestive | Enviromental factors | Lee KJ, 2020 | Greenness of the environment within a 100-meter NDVI buffer | 11 | NA | 0.98 | 0.98 | 0.98 | 0.72 | 0(0-0.51)(0.67) | 0.97-0.99 | 1 | NA | NA | NA | NA |
| Weak | Obstetric history | Wahabi HA, 2020 | Preconception care | 9 | 788/1626 | 0.83 | 0.86 | 1.14 | 0.12 | 0.22(0-0.64)(0.25) | 0.57-1.21 | 1 | 0.05 | 0.05 | 0.71 | 0.52 |
| Weak | Medical history | McDonald SD, 2011 | High gestational weight gain | 3 | 89418/1243160 | 0.76 | 0.72 | 0.73 | 0.68 | 0.16(0-0.77)(0.30) | 0.07-7.65 | 1 | 0 | 0.02 | 1.28 | 0.81 |
| Weak | Assisted reproductive techniques | Grady R, 2017 | Single embryo transfer (randomized clinical trials) | 4 | 388/446 | 0.37 | 0.37 | 0.38 | 0.10 | 0(0-0.68)(0.97) | 0.15-0.87 | 2 | <0.01 | <0.01 | 2.15 | 0.74 |
| Weak | Supplements | Zhang Y, 2020 | Magnesium supplementation | 6 | 728/1264 | 0.59 | 0.68 | 1.07 | 0.10 | 0.54(0-0.80)(0.05) | 0.15-2.23 | 3 | <0.01 | 0.03 | 0.33 | <0.01 |
